# Natural Frequency Analysis of Cluster Dither with Partially Tapered Spokes for Three-Axis Ring Laser Gyroscopes

**DOI:** 10.3390/s26185772

**Published:** 2026-09-11

**Authors:** Cheon Joong Kim, Jun Eon An, Haesung Yu, JunMin Park

**Affiliations:** 1 Agency for Defense Development, Daejeon 34022, Republic of Koreahae0817@add.re.kr (H.Y.); 2 Department of Electronics Engineering, Chungnam National University, Daejeon 34134, Republic of Korea

**Keywords:** cluster dither, dither natural frequency, partially tapered dither spoke, ring laser gyroscope, inertial measurement unit

## Abstract

The ring laser gyroscope (RLG), a representative type of optical gyroscope, exhibits a lock-in region in which very small angular velocity inputs cannot be measured due to backscattering from the mirrors. Generally, this lock-in effect is mitigated by applying a high-amplitude sinusoidal vibration to the gyro body. The mechanical device used to induce this sinusoidal vibration is referred to as a dither. Dithers vary in configuration depending on the size of the gyro; while single-axis dithers are applied to gyros with relatively large optical paths, a cluster dither—which simultaneously applies sinusoidal vibrations to three-axis gyroscopes—is used for those with smaller optical paths. Unlike in the single-axis dither, the gyro body is mounted on the cluster dither at a specific angle. Furthermore, while the dither fixing hole in a single-axis dither is located at the center of the gyro body, it is positioned on the periphery in a cluster dither. Consequently, the center of rotation for a cluster dither is located at the center of the dither itself, rather than coinciding with the center of the gyro body. To increase the natural frequency of the cluster dither with such a configuration, this paper proposes a design employing a partially tapered non-uniform and heterogeneous cantilever beam, rather than the uniform and homogeneous cantilever beam used in conventional single-axis dither spokes, and presents a corresponding natural frequency analysis method. The accuracy of the proposed natural frequency analysis method is validated through modeling and simulation (M&S) and through experimental testing of fabricated prototypes.

## 1. Introduction

A ring laser gyroscope (RLG), a representative optical gyroscope, has a region in which small externally applied angular rates cannot be measured because of backscattering from the mirrors; this region is referred to as lock-in [1,2]. A representative method for solving this lock-in problem is to apply a high-amplitude sinusoidal vibration to the gyro body, thereby minimizing the time during which the externally applied angular rate remains in the lock-in region. Because the sinusoidal vibration applied by the dither can be removed by integrating the gyro output over each dither period, the externally applied angular rate can be measured by using this method. The mechanical device that applies sinusoidal vibration to the gyro body is called a dither, and various dither configurations can be implemented depending on the size of the RLG [1,2,3,4,5]. In a dither-based method, a periodic voltage corresponding to the first-mode natural frequency of the dither is applied to the lead zirconate titanate (PZT) elements attached to the dither, generating a bending moment in the dither structure. Therefore, the main design factors of a dither are its first-mode natural frequency and vibration amplitude. To improve the random-walk noise performance of an RLG by minimizing the time during which the externally applied angular rate remains in the lock-in region, the angular rate of the dither must be increased [2]. One way to increase the dither angular rate for the same angular displacement amplitude is to increase the natural frequency of the dither [3,4,5]. However, when the dither is modeled as a second-order damped system, its angular displacement amplitude is known to be inversely proportional to the stiffness of the dither spoke and proportional to the Q-factor. Therefore, if the Q-factor remains constant, increasing the spoke stiffness increases the dither natural frequency in proportion to the square root of the stiffness, but the angular displacement amplitude decreases more rapidly, resulting in a reduction in the overall angular rate. When the damping characteristics of the dither spoke remain constant, however, the Q-factor is proportional to the dither natural frequency; accounting for this relationship, an increase in the dither natural frequency increases the dither angular rate. Therefore, as the lock-in region of an RLG becomes larger, the natural frequency of the dither must be increased accordingly. However, under actual dither operating conditions, the damping characteristics of the dither spoke may not remain constant; the exact relationship between the dither natural frequency and Q-factor will be examined in future work. An excessive increase in dither natural frequency can also cause problems such as reduced angular displacement amplitude owing to increased stiffness, higher PZT driving voltage, reduced RLG mounting space owing to a smaller dither spoke geometry, increased stress, and reduced fatigue life. Appropriate selection of the dither natural frequency is therefore very important. In general, single-axis dither natural frequencies of approximately 400 Hz for a 28 cm optical path RLG, approximately 600 Hz for a 16 cm optical path RLG, and approximately 1000 Hz or higher for a 6 cm optical path RLG have been reported. Because the lock-in region increases as the optical path of the RLG becomes shorter, the dither should be designed with an increased natural frequency and dither angular rate to reduce random-walk noise, phase error, and quantization noise [2,3,4,5,6].

A single-axis dither is generally mounted at the center of the RLG body and is manufactured so that sinusoidal vibration is applied to the gyro body fixed to the end of the dither spoke [6]. Therefore, a single-axis dither can be modeled as a clamped–free cantilever, and many studies have addressed its design and analysis [7,8,9,10]. When such single-axis dithers are applied to an inertial measurement unit (IMU) equipped with three RLGs, three dithers with different natural frequencies are required, one for each axis [1]. A single-axis dither is commonly used when the optical path of the RLG is relatively large. However, when the optical path becomes smaller, the allowable mounting area for the dither is limited, making dither design and fabrication difficult. To overcome this limitation, a cluster dither capable of simultaneously applying mechanical vibration to three gyro bodies has been proposed [11]. The proposed cluster dither is essential for miniaturized IMU designs based on RLGs. However, only the configuration has been proposed, and no detailed study on the design and analysis of the cluster dither has been reported.

Because three RLGs must be mounted on a cluster dither, three dither spokes are mutually supported at the dither center and are designed to be fixed to the housing at 120° intervals. The rotation axis of the cluster dither is located at the center of the dither, and the three RLGs are mounted in the spaces between the three spokes at 120° intervals around the dither rotation axis, with each RLG inclined by 64° with respect to that axis. One end of each cluster dither spoke is connected to the gyro mounting part at the rotation center of the dither, and the other end is fixed to the IMU housing. Therefore, unlike a single-axis dither modeled as a uniform cantilever beam, the cluster dither should be modeled as a clamped–supported cantilever beam. Since the modeling of a dither depends heavily on its configuration, the natural frequency analysis method must be tailored to the specific design. Compared with the conventional analysis method for a single-axis dither, a cluster dither must be modeled as a clamped–supported cantilever; its boundary conditions are therefore different, its spoke-end mass has a complex structure, and its spoke is non-uniform. A new method for analyzing the natural frequency of the cluster dither is consequently required. In particular, the support condition in this study cannot be represented adequately by the end-displacement condition alone; the moment- and shear-force-equilibrium conditions at the support must also be considered.

As noted above, the natural frequency of a cluster dither is related to the size of the RLG lock-in region. References [3,4,5] designed dithers for 6 cm optical path RLGs with natural frequencies of approximately 1000–1380 Hz and angular rate amplitudes of 500–1000°/s, which are much higher than those of 28 cm or 16 cm optical path RLGs. Because the RLG considered in this study has an optical path of approximately 10 cm, a new dither natural frequency must be designed. The lock-in region of the 10 cm optical path RLG considered in this paper is known to be approximately 0.1–0.125°/s, and the target random-walk noise performance is approximately 0.006 deg/√h. To satisfy this requirement, the dither angular rate should be at least approximately 200–300°/s [2]. To properly apply this angular velocity to the RLG mounted on the cluster dither, it is necessary to account for the 64° tilt angle of the RLG relative to the rotation axis of the cluster dither. Therefore, the actual applied velocity must be 450–700°/s, which is obtained by dividing 200–300°/s by cos 64°. Based on the design conditions for dither frequency and angular rate amplitude according to the RLG optical path length, the required natural frequency of the cluster dither was set to 800 Hz or higher, and the cluster dither was designed to satisfy this requirement. The method proposed in this paper to increase the natural frequency of the cluster dither is to make the dither spoke partially tapered from the fixed part along the spoke length, rather than increasing the overall spoke thickness. This approach provides additional mounting space for the RLGs between the dither spokes while simultaneously increasing the natural frequency of the dither. Because this design changes the conventional uniform and homogeneous cantilever-type spoke used in single-axis dithers into a composite cross-section cantilever-type spoke, a new natural frequency analysis method is required; this paper presents that method.

Euler–Bernoulli beam theory and the Rayleigh method, which are widely used for conventional beam structures, are well established. However, if the dynamic equilibrium conditions at the supported end are not properly incorporated, directly applying these methods to the cluster dither considered in this study can lead to significant analytical errors. In particular, the simplified cantilever beam models with a concentrated tip mass presented in References [6,12] mainly address clamped–free structures. Therefore, they do not explicitly account for the coupling between moment and shear-force equilibrium at the supported end. The cluster dither investigated in this study has a clamped–supported configuration with partially tapered dither spokes and an eccentric tip mass. Therefore, accurate analysis of its natural frequencies requires not only consideration of the structural nonuniformity and the characteristics of the tip mass, but also proper implementation of the moment and shear-force equilibrium conditions at the supported end. The pronounced differences in natural frequency resulting from different formulations of the boundary conditions are examined in Section 4 through comparison with M&S results.

Consequently, the main contribution of this study is not the development of a new beam vibration theory. Rather, it lies in defining dynamic end boundary conditions appropriate for the cluster dither structure and in formulating and validating a natural-frequency analysis method that incorporates these conditions.

In this paper, the partially tapered dither spoke described above is modeled as a clamped–supported cantilever beam, and its natural frequency is analyzed theoretically. To verify the accuracy of the proposed analysis method, the results are compared with modeling and simulation (M&S) results obtained using ANSYS 2023, a commercial finite element analysis program. Furthermore, by comparing the measured natural frequency of the actual fabricated cluster dither with the theoretical predictions, we verified the validity of the natural frequency analysis model presented in this paper.

## 2. Natural Frequency Analysis of a Single-Axis Dither

### 2.1. Natural Frequency Analysis of a Uniform and Homogeneous Cantilever Beam

**Figure 1:**
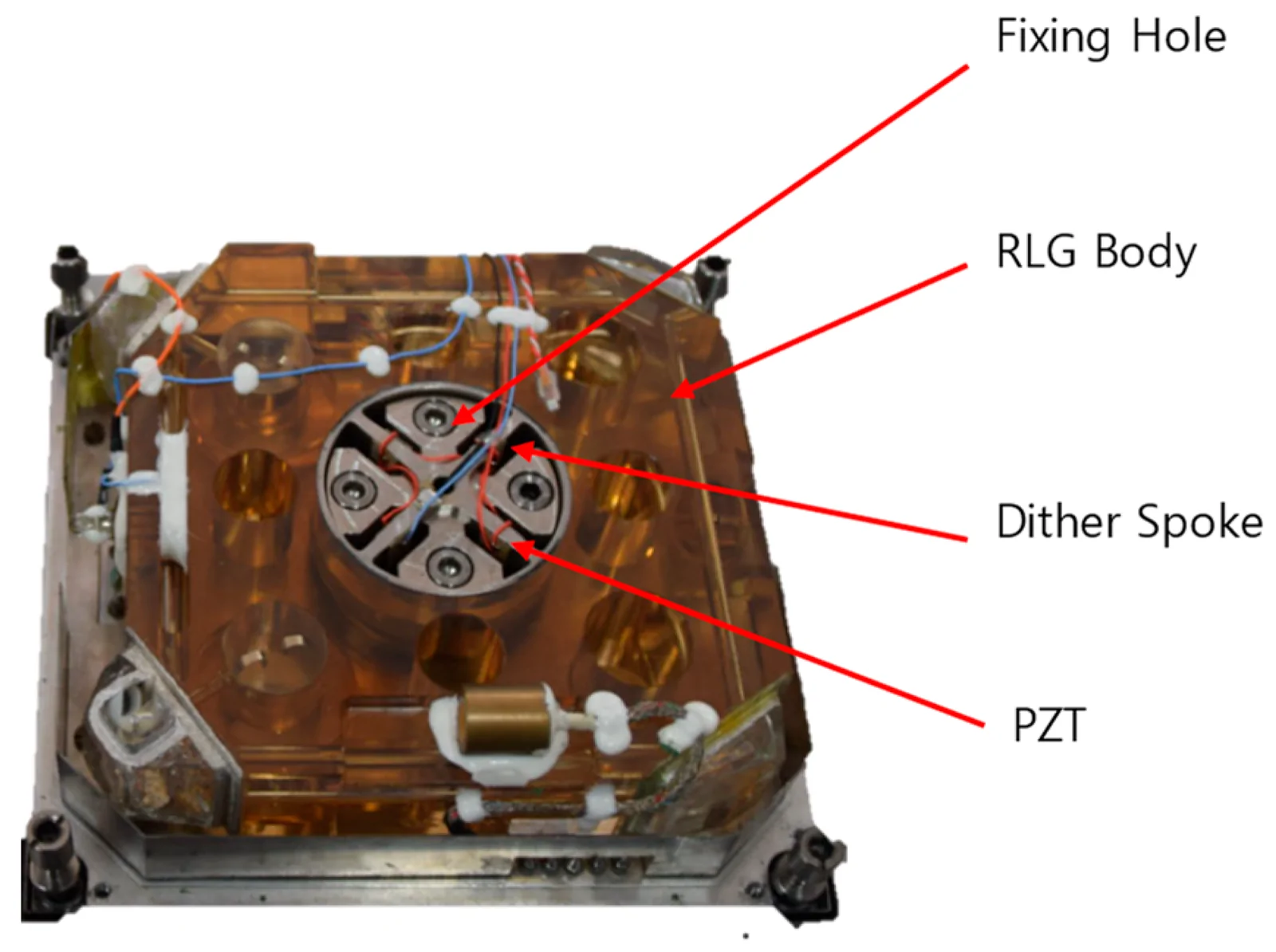
Photograph of a typical single-axis dither.

**Figure 2:**
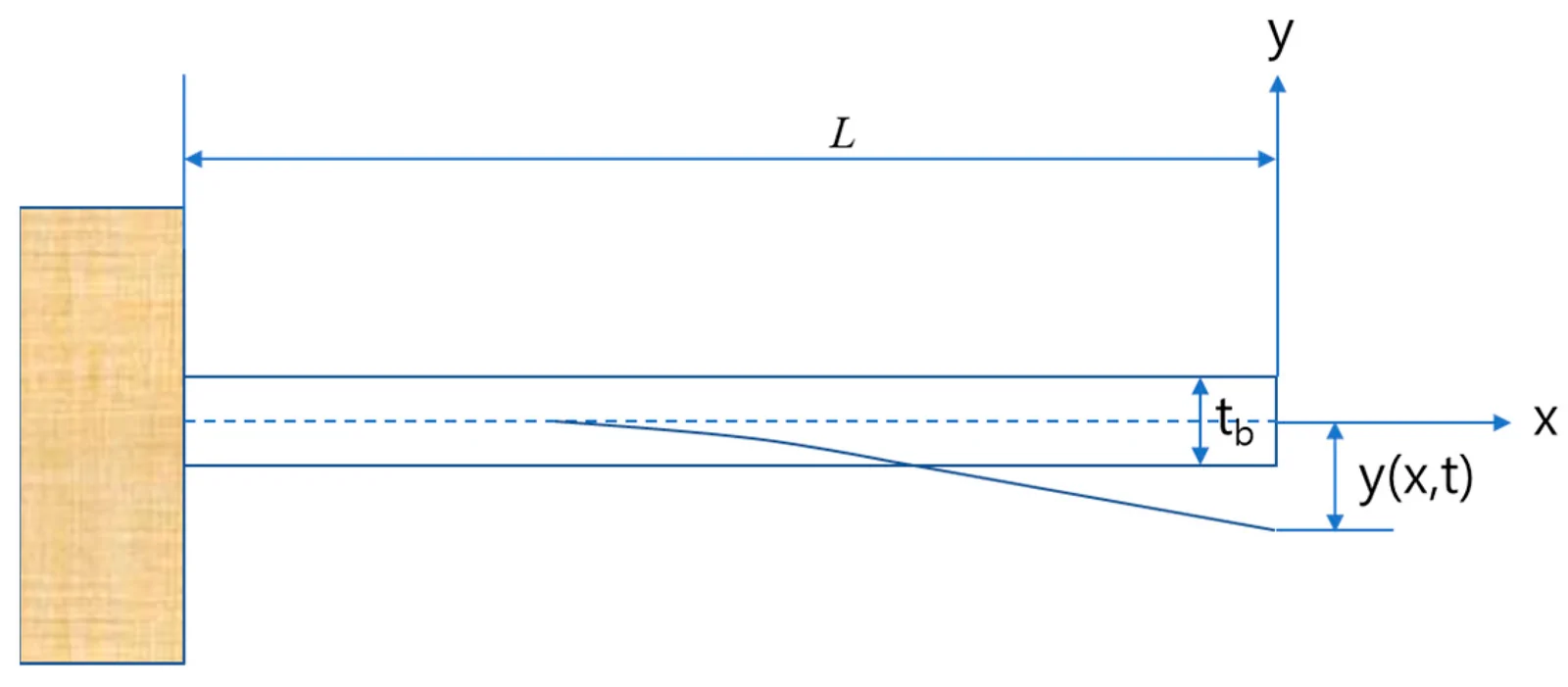
Configuration of a cantilever beam.

A typical single-axis dither is shown in Figure 1 [13]. As shown in Figure 1, a single-axis dither is fixed to the RLG housing by four holes or multiple holes, and the RLG body is fixed to the dither spoke [6,13]. Therefore, when voltage is applied to the PZT attached to the dither spoke, the gyro body undergoes sinusoidal vibration. Because the dither operates according to this principle, the dither spoke can be modeled as the uniform and homogeneous cantilever beam shown in Figure 2. To analyze the natural frequency of the cantilever beam, the equation of motion for transverse deflection is derived as Equation (1) [6,14,15].

Because the dither spoke is a uniform and homogeneous cantilever beam, Young’s modulus *E*, moment of inertia *I*, density *ρ*, and cross-sectional area *A* in Equation (1) are constants, and p(*x*,*t*) is the externally applied force. Because the natural frequency of the cantilever is independent of the external force, p(*x*,*t*) in Equation (1) can be assumed to be zero. Assume that the transverse displacement *y*(*x*,*t*) of the cantilever undergoes sinusoidal vibration as expressed in Equation (2), with natural angular frequency ω_n_ and transverse-displacement amplitude *Y*(*x*). The transverse-displacement amplitude *Y*(*x*) represents the deformation shape of the cantilever along its length and is also called the mode shape function. Applying separation of variables to *y*(*x*,*t*), deriving the characteristic equation with respect to *x*, and obtaining its eigenvalue gives Equation (3); the corresponding general solution is given by Equation (4). To determine the coefficients of the general solution in Equation (4), the boundary conditions shown in Equation (5) are required. These boundary conditions mean that, at the fixed support (*x* = 0), both the transverse displacement and its slope are zero, whereas at the free end (*x* = *L*), the bending moment and shear force are also zero [6,14,15]. Applying the *x* = 0 boundary conditions to Equation (4), solving for the coefficients that satisfy them, and substituting the coefficients into Equation (4) gives Equation (6). Applying the *x* = *L* boundary conditions to Equation (6) gives Equation (7). The values of λL that make the determinant of Equation (7) zero form periodic solutions, and the first solution is 0.59686π. Rearranging Equation (3) with respect to *f_n_* gives Equation (8), and substituting the value of λL satisfying Equation (7) into Equation (8) gives the natural frequency *f_n_* of the cantilever beam.

(1) $$EI\frac{\partial^{4}y}{\partial x^{4}}+\rho A\frac{\partial^{2}y}{\partial t^{2}}=p(x,t)$$

(2) $$y(x,t)=Y(x)\cos(\omega_{n}t+\psi )$$

(3) $$\lambda^{4}=\frac{\rho A\omega_{n}^{2}}{EI}$$

(4) $$Y(x)=c_{1}\sin(\lambda x)+c_{2}\cos(\lambda x)+c_{3}\sinh(\lambda x)+c_{4}\cosh(\lambda x)$$

(5) $$Y(0)=0,\;\frac{dY(0)}{dx}=0,\;\frac{d^{2}Y(L)}{dx^{2}}=0,\;\frac{d^{3}Y(L)}{dx^{3}}=0$$

(6) $$Y(x)=c_{1}(\sin(\lambda x)-\sinh(\lambda x))+c_{3}(\cos(\lambda x)-\cosh(\lambda x))$$

(7) $$\left[\begin{array}{c} \begin{array}{cc} -\lambda^{2}(\sin(\lambda L)+\sinh(\lambda L)) & -\lambda^{2}(\cos(\lambda L)+\cosh(\lambda L)) \end{array}\\ \begin{array}{cc} -\lambda^{3}(\cos(\lambda L)+\cosh(\lambda L)) & \lambda^{3}(\sin(\lambda L)-\sinh(\lambda L)) \end{array} \end{array}\right]\left[\begin{array}{c} c_{1}\\ c_{3} \end{array}\right]=0$$

(8) $$f_{n}=\frac{1}{2\pi }{\left(\lambda L\right)}_{n}^{2}\sqrt{\frac{EI}{mL^{3}}}$$

In Equation (7), *m* is the mass (ρAL) of the dither spoke. A beam can have various boundary conditions depending on how its fixed and end supports are constrained. For the beam in Figure 2, when the left end is clamped and the right end is supported, the boundary conditions are given by Equation (9). Substituting Equation (4) into the boundary conditions of Equations (5) and (9), deriving an equation analogous to Equation (7), and obtaining the first value of λL that makes the determinant zero gives the results summarized in Table 1 [14]. Table 1 shows that the natural frequency varies greatly with the beam boundary conditions. As stated in the introduction, a single-axis dither can be modeled as a clamped–free cantilever, whereas a cluster dither can be modeled as a clamped–supported cantilever, as indicated in Table 1. The results in Table 1 demonstrate that the conventional natural frequency analysis method for a single-axis dither cannot be applied directly to a cluster dither.

(9) $$Y(0)=0,\;\frac{dY(0)}{dx}=0,\;Y(L)=0,\;\frac{d^{2}Y(L)}{dx^{2}}=0$$

### 2.2. Natural Frequency Analysis of a Uniform and Homogeneous Cantilever Beam with an End Mass

**Figure 3:**
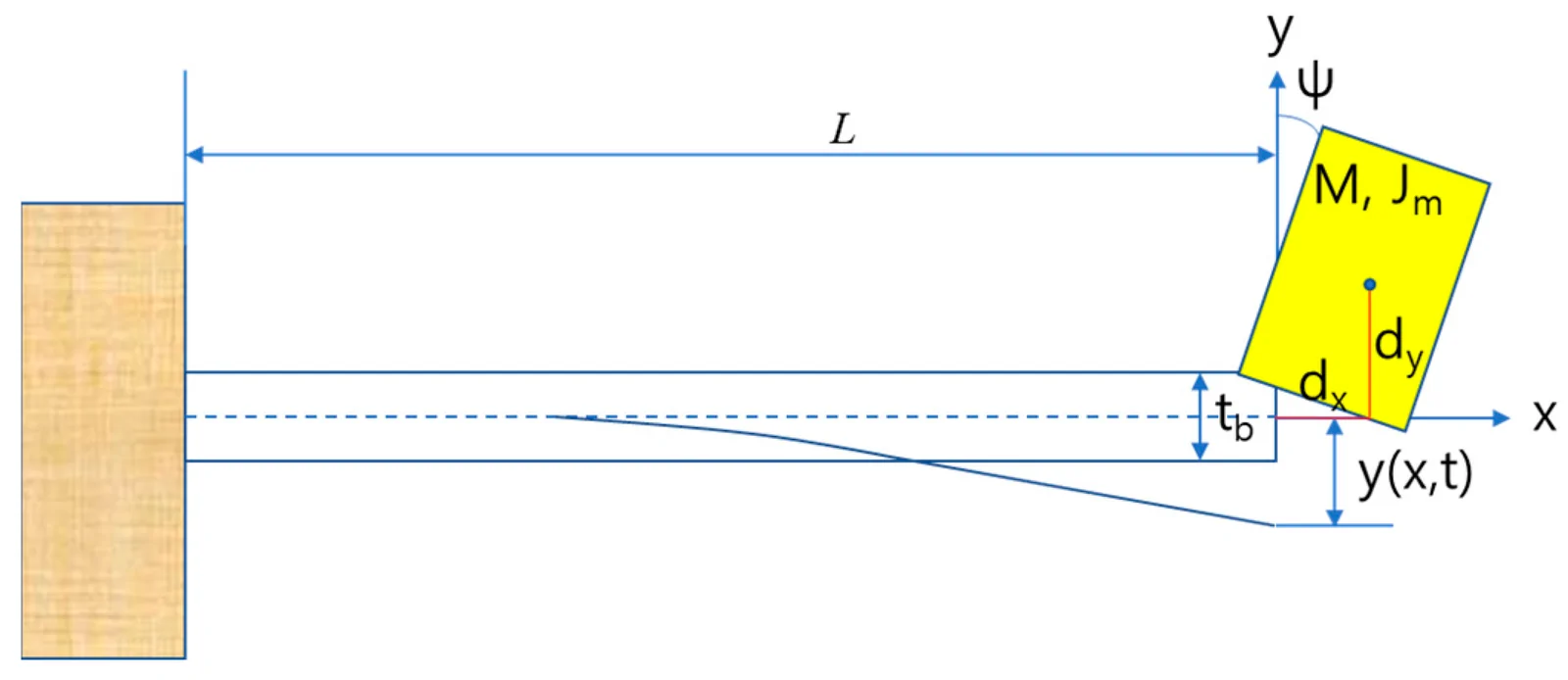
Configuration of a cantilever beam with an end mass.

Section 2.1 derived the natural frequency formula for a uniform and homogeneous cantilever beam without an attached end mass. This section derives the equation of motion for the case in which an object with mass *M* and moment of inertia *J_m_* is attached to the end of the cantilever beam, as shown in Figure 3. The center of mass of the attached object is assumed to be offset from the end of the beam by *d_x_* in the x direction, *d_y_* in the y direction, and *d_z_* in the z direction. In addition, considering that the gyro body is mounted with an inclination relative to the rotation axis of the cluster dither, the object attached to the end of the beam is assumed to be mounted after a rotation of -*ψ* about the z axis, as shown in Figure 3.

To calculate the natural frequency of a clamped–free cantilever beam with a rotated end mass, as shown in Figure 3, a new equation must be derived. The beam itself is identical to that in Figure 2, so Equations (1)–(5) can be applied without change. However, because an object is attached to the beam end, the boundary conditions at *x* = *L* are different; thus, the boundary condition in Equation (5) must be newly derived. The boundary conditions at *x* = *L* are derived by assuming that the transverse bending moment and shear force generated by the end mass are equal to the bending moment and shear force of the beam itself. This equilibrium of shear force and bending moment at the beam end is expressed as Equations (10) and (11) [12,16,17]. Substituting Equation (2) into Equations (10) and (11) gives the transverse-displacement boundary conditions shown in Equations (12) and (13).

(10) $$EI\frac{\partial^{3}y(L,t)}{\partial x^{3}}-M\frac{\partial^{2}y(L,t)}{\partial t^{2}}-Md_{x}\frac{\partial^{3}y(L,t)}{\partial t^{2}\partial x}=0$$

(11) $$EI\frac{\partial^{2}y(L,t)}{\partial x^{2}}+J_{zz}\frac{\partial^{3}y(L,t)}{\partial t^{2}\partial x}+Md_{x}\frac{\partial^{2}y(L,t)}{\partial t^{2}}=0$$

(12) $$EI\frac{d^{3}Y(L)}{dx^{3}}+M\omega_{n}^{2}Y(L)+Md_{x}\omega_{n}^{2}\frac{dY(L)}{dx}=0$$

(13) $$EI\frac{d^{2}Y(L)}{dx^{2}}-J_{zz}\omega_{n}^{2}\frac{dY(L)}{dx}-Md_{x}\omega_{n}^{2}Y(L)=0$$

Here, *M* is the mass of the end object, and *J_zz_* is the moment of inertia of the end object about the *z* axis with respect to the beam-end axis. However, when the center of mass of the end object is offset from the beam end by the distance vector *d* = [*d_x_ d_y_ d_z_*] and the object has rotations *φ*, *θ*, and *ψ* about the x, y, and z axes, respectively, the moment of inertia about the mounting axis of the end object is calculated using the parallel-axis theorem and the coordinate transformation matrix, as shown in Equation (14) [14]. In Equation (14), *J_m_* is the inertia matrix of the attached end object, *d* is the offset vector from the beam end to the center of mass of the attached object, and *I*_3*3_ is the identity matrix. Substituting Equations (8) and (14) into Equations (12) and (13) and substituting the displacement expression of Equation (6), which satisfies the boundary conditions at *x* = *L*, into Equations (12) and (13), gives Equation (15) in terms of the coefficients *c*_1_ and *c*_3_ of Equation (6). The variables composing Equation (15) are summarized in Equations (16)–(18). The natural frequency of the cantilever beam in Figure 3 can be calculated by finding the value of λL that makes the determinant of Equation (15) zero, as in the method presented in Section 2.1, and substituting it into Equation (8).

(14) $$J=C_{m}^{b}J_{m}C_{b}^{m}+M(dd^{T}I_{3\times 3}-d^{T}\cdot d)$$

(15) $$\left[\begin{array}{c} \begin{array}{cc} -G_{2}(L)+\mu_{M}(\lambda L)F_{1}(L)+\mu_{d}{\left(\lambda L\right)}^{2}F_{2}(L) & F_{1}(L)+\mu_{M}(\lambda L)F_{2}(L)-\mu_{d}{\left(\lambda L\right)}^{2}G_{1}(L) \end{array}\\ \begin{array}{cc} -G_{1}(L)-\mu_{J}{\left(\lambda L\right)}^{3}F_{2}(L)-\mu_{d}{\left(\lambda L\right)}^{2}F_{1}(L) & -G_{2}(L)+\mu_{J}{\left(\lambda L\right)}^{3}G_{1}(L)-\mu_{d}{\left(\lambda L\right)}^{2}F_{2}(L) \end{array} \end{array}\right]\left[\begin{array}{c} c_{1}\\ c_{3} \end{array}\right]=0$$

(16) $$F_{1}(x)=\sin(\lambda x)-\sinh(\lambda x),\;F_{2}(x)=\cos(\lambda x)-\cosh(\lambda x)$$

(17) $$G_{1}(x)=\sin(\lambda x)+\sinh(\lambda x),\;G_{2}(x)=\cos(\lambda x)+\cosh(\lambda x)$$

(18) $$\mu_{M}=\frac{M}{\rho AL},\;\mu_{d}=\frac{M_{d}}{\rho AL^{2}},\;\mu_{J}=\frac{J}{\rho AL^{3}}$$

So far, this paper has derived a method for calculating the natural frequency of a single-axis dither by obtaining λL from the end boundary conditions for a cantilever beam with an attached end object and substituting it into Equation (8). Other methods for calculating the natural frequency of a single-axis dither have also been reported. Reference [6] likewise models the dither as a uniform and homogeneous cantilever beam, but treats the dither spoke and the RLG attached to the spoke end as point masses on the spoke and calculates the natural frequency using their relationship with the moment of inertia. Reference [6] reported that the natural frequency analysis differed by approximately 2% from the M&S and experimental results for a fabricated dither.

Reference [12] presented two methods for a uniform and homogeneous cantilever beam with an end object of mass M and moment of inertia *J_m_*, as shown in Figure 3. The first substitutes Equation (4) into the end boundary conditions of Equations (12) and (13), derives an equation similar to Equation (15), and obtains the natural frequency from the point at which its determinant becomes zero. The second obtains the natural frequency directly using the Rayleigh method. Reference [12] reported that the first-mode natural frequency obtained analytically agreed with that obtained using an M&S tool to within 1%, demonstrating highly accurate analysis. The natural frequency analysis methods in References [6,12] model the dither as a uniform and homogeneous cantilever beam, assume a simple structure for the mass attached to the dither spoke end, and use boundary conditions different from those of a cluster dither. These methods are therefore difficult to apply directly to the natural frequency analysis of a cluster dither, and a new natural frequency analysis method is required.

## 3. Configuration Design and Natural Frequency Analysis of a Cluster Dither with Partially Tapered Spokes

**Figure 4:**
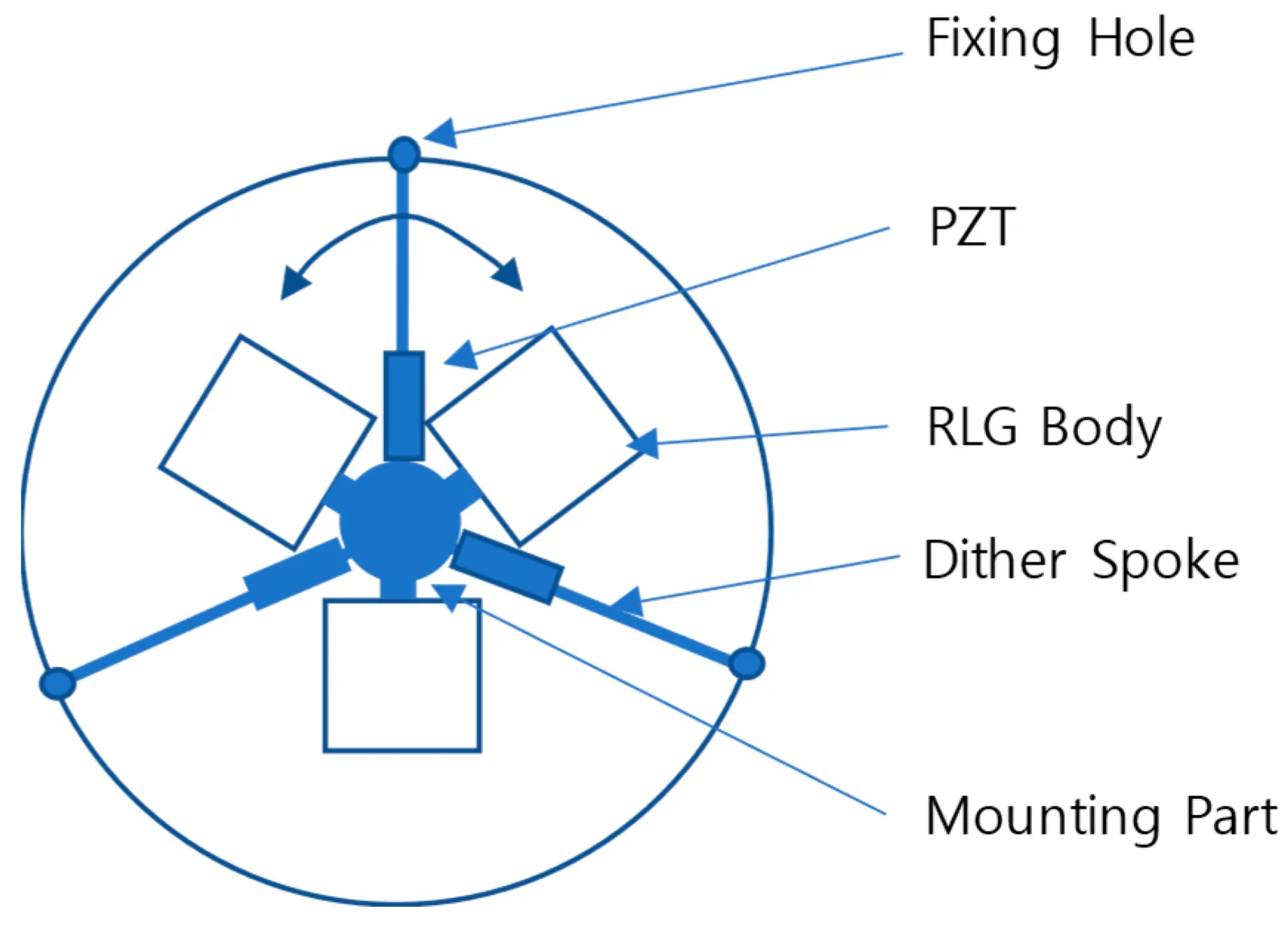
Configuration of the cluster dither.

As shown in Figure 1, a single-axis dither has one gyro body fixed to the ends of multiple dither spokes, and the rotation center of the dither coincides with the center of the fixed part. A cluster dither has a different configuration from a single-axis dither because it must simultaneously apply sinusoidal vibration to the three gyro bodies that constitute an IMU. As one example of a cluster dither, Reference [11] proposed a configuration in which three gyro bodies are inclined at 120° intervals in the form of a tetrahedral pyramid and mounted at the end of each spoke. Based on the cluster dither configuration proposed in Reference [11], this section presents the design of a cluster dither considering the size of the mounted gyro, together with a natural frequency analysis method for a design having a natural frequency of 800 Hz or higher. The schematic configuration of the cluster dither is shown in Figure 4.

**Figure 5:**
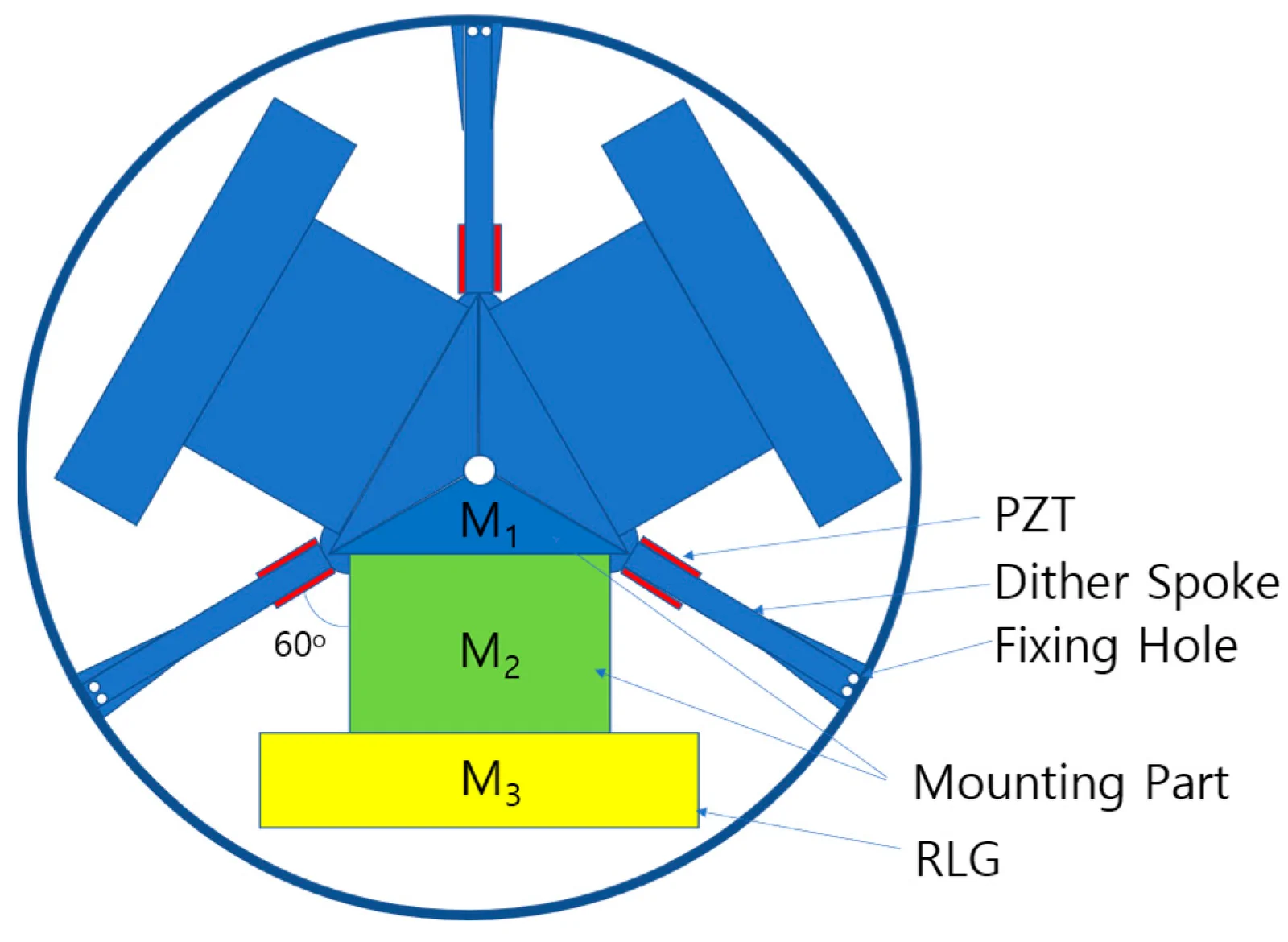
Overall top view of the cluster dither.

**Figure 6:**
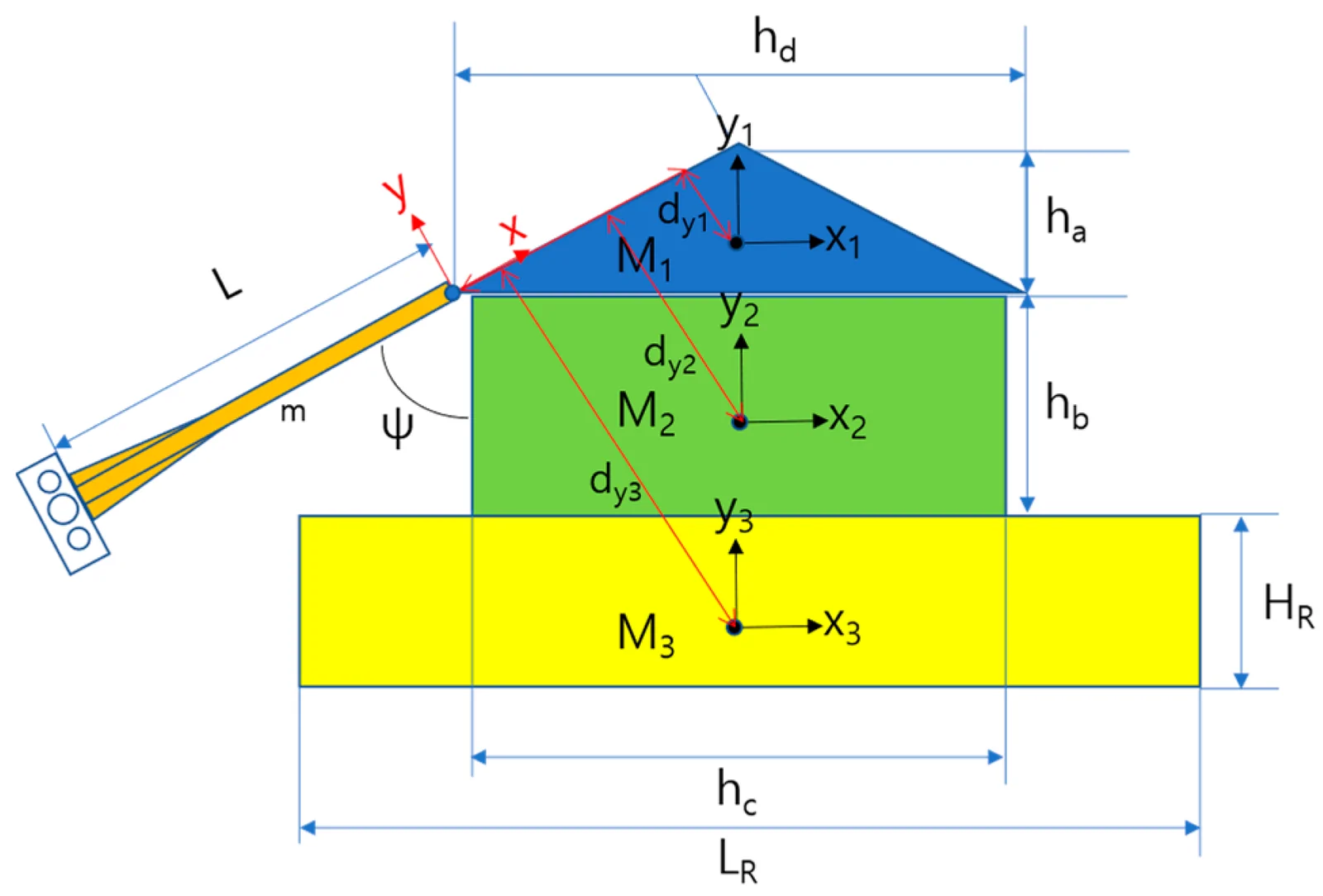
Top view of the spoke, mounting part, and RLG in the cluster dither.

**Figure 7:**
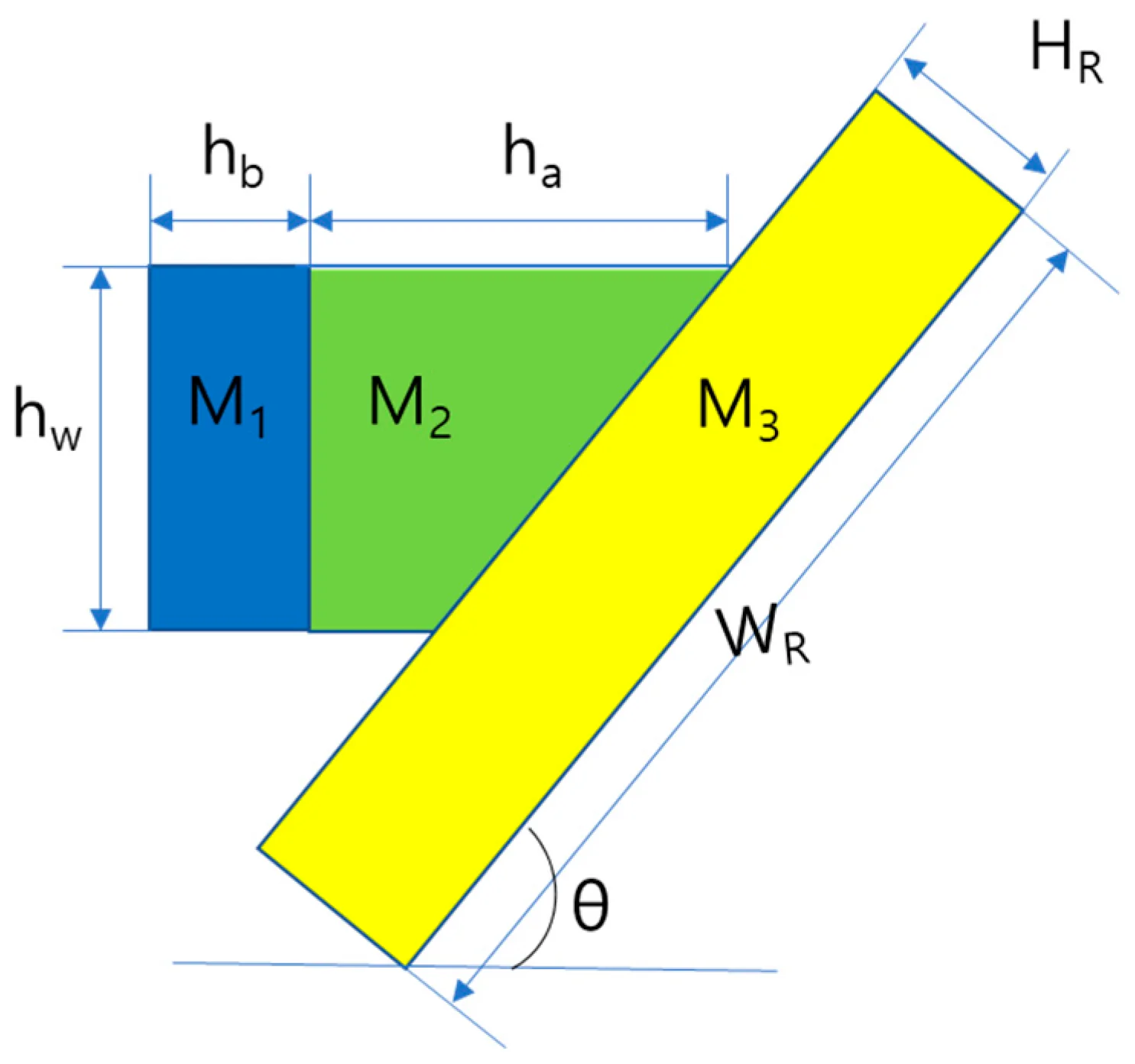
Side view of mounting part and RLG in the cluster dither.

The size of the cluster dither was determined considering the fixed outer diameter of the IMU to which it is applied. The cluster dither has three spokes for mounting three gyro bodies, and mounting parts were added to the ends of the spokes to accommodate small RLGs with an optical path of approximately 10 cm. The gyro mounting parts were designed in a rectangular shape for ease of machining. As described above, the gyros mounted on the cluster dither were designed to be inclined at 120° intervals, and the cluster dither was designed to be fixed to the IMU housing by six screws. A voltage corresponding to the natural frequency of the dither is periodically applied to the PZT elements fixed to the spokes. The PZT elements were attached close to the rotation center of the dither to maximize the dither amplitude. A schematic top view of the designed cluster dither is shown in Figure 5. In Figure 5, the gyro mounting part consists of *M*_1_, which mutually supports the dither, and *M*_2_, which mounts the gyro, while *M*_3_ is the RLG. To satisfy the natural frequency requirement of 800 Hz or higher, the dither spoke was designed to be partially tapered from the fixed part toward the rotation center so that the effective Young’s modulus of the spoke could be increased and the natural frequency adjusted to the required value. As shown in Figure 5, the cluster dither consists of three identical structures mutually supported at the rotation center; therefore, the natural frequency of the entire cluster dither can be obtained by analyzing one dither structure. For this purpose, the detailed modeling of the gyro mounting part and gyro at the end of one spoke is shown in Figure 6 and Figure 7.

**Figure 8:**
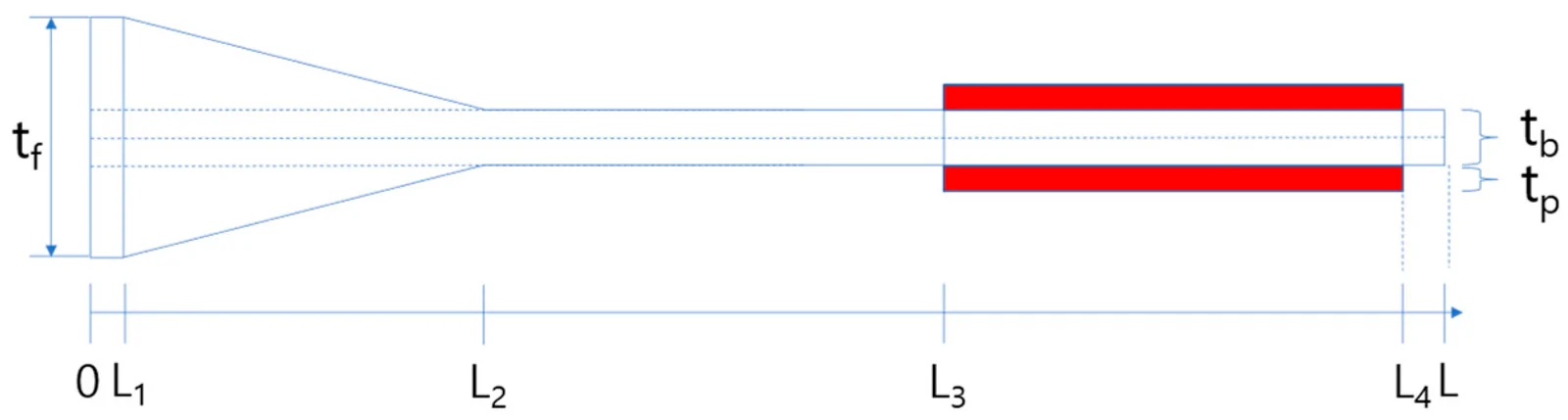
The top view of dither spoke in the cluster dither.

Figure 8 shows the detailed design of the dither spoke in Figure 5. Because the cluster dither spoke designed in this paper is partially tapered and a PZT is attached to the spoke, the natural frequency formula for a uniform and homogeneous cantilever beam derived in Section 2 cannot be applied directly. A new derivation is therefore required. First, the equation of motion for a non-uniform and heterogeneous cantilever beam is derived as Equation (19). Multiplying both sides of Equation (19) by Equation (2) and integrating over the entire length of the dither spoke gives Equation (20). Rearranging the integral in the first term of Equation (20) gives Equation (21). When the clamped–supported cantilever boundary conditions in Equation (9) are substituted into Equation (21), the first term becomes zero; substituting this result into Equation (20) and rearranging with respect to the natural frequency gives Equation (22). Transforming Equation (22) in terms of λL, equivalent stiffness, and equivalent mass per unit length gives Equation (23). Equation (23) has the same form as Equation (8), because it converts the non-uniform and heterogeneous cantilever beam into a uniform and homogeneous cantilever beam by calculating its equivalent stiffness and equivalent mass per unit length. For Equation (23), λL, equivalent stiffness, and equivalent mass per unit length are expressed in Equations (24)–(26) [18]. Equations (24)–(26) show that the partially tapered dither spoke in Figure 8 can be modeled as a uniform and homogeneous cantilever beam having equivalent stiffness and equivalent mass per unit length, rather than being treated directly as a non-uniform and heterogeneous beam.

(19) $$\frac{\partial^{2}}{\partial x^{2}}E(x)I(x)\frac{\partial^{2}y(x,t)}{\partial x^{2}}+\rho (x)A(x)\frac{\partial^{2}y(x,t)}{\partial t^{2}}=0$$

(20) $$\cos(\omega_{n}t+\psi )\int_{0}^{L}Y(x)\frac{d^{2}}{dx^{2}}E(x)I(x)\frac{d^{2}Y(x)}{dx^{2}}dx-\omega_{n}^{2}\cos(\omega_{n}t+\psi )\int_{0}^{L}Y(x)\rho (x)A(x)Y(x)dx=0$$

(21) $$\int_{0}^{L}Y(x)\frac{d^{2}}{dx^{2}}E(x)I(x)\frac{d^{2}Y(x)}{dx^{2}}dx={\left[\left(\frac{d}{dx}E(x)I(x)\frac{d^{2}Y(x)}{dx^{2}}Y(x)\right)-\left(E(x)I(x)\frac{d^{2}Y(x)}{dx^{2}}\right)\frac{dY(x)}{dx}\right]}_{0}^{L}+\int_{0}^{L}E(x)I(x)\frac{d^{2}Y(x)}{dx^{2}}\frac{d^{2}Y(x)}{dx^{2}}dx$$

(22) $$\omega_{n}^{2}=\frac{\int_{0}^{L}E(x)I(x){\left(\frac{d^{2}Y(x)}{dx^{2}}\right)}^{2}dx}{\int_{0}^{L}\rho (x)A(x)Y^{2}(x)dx}$$

(23) $$f_{n}=\frac{1}{2\pi }{\left(\lambda L\right)}_{n}^{2}\sqrt{\frac{{\left(EI\right)}_{eq}}{{\left(\rho A\right)}_{eq}L^{4}}}$$

(24) $$\lambda^{4}=\frac{\int_{0}^{L}{\left(\frac{d^{2}Y(x)}{dx^{2}}\right)}^{2}dx}{\int_{0}^{L}Y^{2}(x)dx}$$

(25) $${\left(EI\right)}_{eq}=\frac{\int_{0}^{L}E(x)I(x){\left(\frac{d^{2}Y(x)}{dx^{2}}\right)}^{2}dx}{\int_{0}^{L}{\left(\frac{d^{2}Y(x)}{dx^{2}}\right)}^{2}dx}$$

(26) $${\left(\rho A\right)}_{eq}=\frac{\int_{0}^{L}\rho (x)A(x)Y^{2}(x)dx}{\int_{0}^{L}Y^{2}(x)dx}$$

As discussed in Section 2, the boundary conditions of the cluster dither are required to obtain the coefficient λL in Equation (23). The spoke of a single-axis dither was modeled as a cantilever beam in Section 2, but the spoke of a cluster dither must be modeled as a clamped–supported cantilever because, as shown in Figure 4, the three dither spokes mutually support each other at the rotation center. In a clamped–supported cantilever, the boundary conditions at *x* = *L* differ from those of a cantilever and must therefore be newly derived. The cantilever boundary conditions in Equations (10) and (11) assume equilibrium of shear force and moment at the end of the beam. However, for a clamped–supported cantilever, the end can rotate but is nearly fixed in displacement; thus, the condition *Y*(*L*) = 0 in Equation (9) holds. Therefore, the second time derivative of *Y*(*L*) is also zero. Substituting this condition into Equations (10) and (11) gives the newly derived boundary conditions of the clamped–supported cantilever, as shown in Equations (27) and (28). Substituting Equation (6) into Equations (27) and (28) gives Equation (29).

(27) $${\left(EI\right)}_{eq}\frac{\partial^{3}y(L,t)}{\partial x^{3}}-Md_{x}\frac{\partial^{3}y(L,t)}{\partial t^{2}\partial x}=0$$

(28) $${\left(EI\right)}_{eq}\frac{\partial^{2}y(L,t)}{\partial x^{2}}+J_{zz}\frac{\partial^{3}y(L,t)}{\partial t^{2}\partial x}=0$$

(29a) $$\left[\begin{array}{c} \begin{array}{cc} -G_{2}(L)+\mu_{d\_eq}{\left(\lambda L\right)}^{2}F_{2}(L) & F_{1}(L)-\mu_{d\_eq}{\left(\lambda L\right)}^{2}G_{1}(L) \end{array}\\ \begin{array}{cc} -G_{1}(L)-\mu_{J\_eq}{\left(\lambda L\right)}^{3}F_{2}(L) & -G_{2}(L)+\mu_{J\_eq}{\left(\lambda L\right)}^{3}G_{1}(L) \end{array} \end{array}\right]\left[\begin{array}{c} c_{1}\\ c_{3} \end{array}\right]=0$$

(29b) $$\mu_{d\_eq}=\frac{M_{d}}{{\left(\rho A\right)}_{eq}L^{2}},\;\mu_{J\_eq}=\frac{J}{{\left(\rho A\right)}_{eq}L^{3}}$$

For the cluster dither modeled as a clamped–supported cantilever, the natural frequency can be calculated by finding the value of λL that makes the determinant of Equation (29a) zero and substituting it into Equation (23), thereby obtaining the natural frequency of a clamped–supported cantilever with an attached end mass.

To analyze the natural frequency of the cluster dither considering the object attached to the end of the dither spoke in Figure 6 and Figure 7, the coefficients required in Equations (25)–(28) must be calculated. First, the mass *m_a_*_1_ and moment of inertia *J_zz_*_1_ of the *M*_1_ model are obtained using Equations (30) and (31), and the mass and moment of inertia of the *M*_2_ model are obtained using Equations (32) and (33). The *M*_3_ model is the RLG and is made of a material different from that of the dither. The RLG has a hexahedral shape with wide upper and lower surfaces and narrow side surfaces, but many optical components are attached to its exterior; these were considered when estimating its mass. The total mass of the *M*_3_ model, including the mass *m_b_* of the bolts for fixing the RLG to the mounting part, is given by Equation (34). The moment of inertia of the RLG is obtained by assuming the RLG to be a hexahedron, calculating the moments of inertia about each axis, and then rotating them by *ψ* about the z axis and by *θ* about the y axis using Equation (14), as shown in Equation (35). The coefficients *M* and *J_zz_* in Equations (27) and (28) are calculated as the sums of the masses and moments of the *M*_1_, *M*_2_, and *M*_3_ models in Figure 6 and Figure 7, as expressed in Equation (36).

(30) $$m_{a1}=\frac{\rho_{b}h_{a}h_{d}h_{w}}{2}$$

(31) $$J_{zz1}=\frac{m_{a1}}{18}\left(h_{a}^{2}+\frac{3}{4}h_{d}^{2}\right)+m_{a1}\left(d_{x1}^{2}+d_{y1}^{2}\right)$$

(32) $$m_{a2}=\frac{\rho_{b}h_{c}h_{w}}{2}\left(h_{a}-\frac{h_{w}}{\tan\psi }\right)$$

(33) $$J_{zz2}=\frac{m_{a2}}{12}(h_{b}^{2}+h_{c}^{2})+m_{a2}(d_{x2}^{2}+d_{y2}^{2})$$

(34) $$m_{a3}=\rho_{R}L_{R}W_{R}H_{R}+m_{b}$$

(35) $$J_{zz3}=\frac{m_{a3}}{12}\left(\left(L_{R}^{2}+H_{R}^{2}\right)\sin^{2}(\theta )\cos^{2}(\psi )+\left(H_{R}^{2}+W_{R}^{2}\right)\sin^{2}(\theta )\sin^{2}(\psi )+\left(L_{R}^{2}+W_{R}^{2}\right)\cos^{2}(\theta )\right)+m_{a3}\left(d_{x3}^{2}+d_{y3}^{2}\right)$$

(36) $$J_{zz}=\sum_{i=1}^{3}J_{zzi},\;M=\sum_{i=1}^{3}m_{ai}$$

As shown in Figure 8, the dither spoke is partially tapered to increase the dither natural frequency, and it is a non-uniform and heterogeneous cantilever beam assembled from components of different materials. Therefore, to model it as a uniform and homogeneous cantilever beam and calculate its natural frequency, the equivalent stiffness and equivalent mass per unit length in Equations (25) and (26) must be calculated. When dissimilar materials are bonded and their shapes are uniform, as in the *L*_3_–*L*_4_ section of Figure 8, the equivalent Young’s modulus can be calculated using Equation (37). In Equation (37), *E_b_* and *E_p_* are the Young’s moduli of the dither spoke and PZT, respectively, and *t_p_* and *t_b_* are the thicknesses of the PZT and the dither spoke. *I_b_* and *I_p_* are the moments of inertia of the dither spoke and PZT in the *L*_3_–*L*_4_ section, respectively, and *I_t_* is the total moment of inertia in that section, as expressed in Equation (38). Using Equations (37) and (38), the equivalent Young’s modulus in the *L*_3_–*L*_4_ section is obtained as Equation (39). All sections other than *L*_3_–*L*_4_ are made of the same material and therefore have a Young’s modulus *E_b_*, whereas the Young’s modulus of the *L*_3_–*L*_4_ section is given by Equation (39). The Young’s modulus of every section of the dither spoke in Figure 8 is thus obtained and is used to calculate the equivalent stiffness in Equation (25).

To calculate the equivalent stiffness and equivalent mass per unit length in Equations (25) and (26), the thickness and mass of each section of the dither spoke in Figure 8 are required in addition to the Young’s modulus of each section; these are summarized in Table 2. In Table 2, h denotes the depth of the dither spoke, and *ρ_b_* and *ρ_p_* denote the densities of the dither spoke and PZT, respectively. Once the thickness and depth of each section listed in Table 2 are known, the moment of inertia of each section can be calculated using Equation (40). Substituting the Young’s moduli and moments of inertia of the dither spoke sections in Figure 8, given by Equations (37)–(39), and the mass per unit length in Table 2 into Equations (24) and (25) gives the equivalent stiffness and equivalent mass per unit length shown in Equations (41) and (42). In Equations (41) and (42), *Y*(*x*) is the transverse-displacement amplitude or mode shape function. Its general solutions are given by Equations (4) and (6); however, because of the computational difficulty noted above, *Y*(*x*) is generally modeled using a polynomial or a low-order transcendental function satisfying the cantilever boundary conditions. Many studies have examined mode shape function modeling methods and their accuracy [19,20]. In this paper, based on Reference [19], *Y*(*x*) is modeled using the fourth-order polynomial in Equation (43). Reference [19] demonstrated that, for a cantilever subjected to a uniformly distributed load, a fourth-order or higher polynomial provides a more accurate mode-shape approximation for first-mode analysis. Because Equation (43) is an admissible rather than an exact shape function, it is suitable for calculating the first-mode natural frequency, but its accuracy deteriorates when it is applied to the second-mode natural frequency. FEM or a more sophisticated analytical method is therefore required for second-mode analysis. The coefficients of Equation (43) are obtained using the clamped–supported cantilever boundary conditions in Equation (9). However, the end-displacement condition *Y*(*L*) = 0 in Equation (9) is expected to hold only when the three cluster dither spokes are manufactured perfectly identically, their end masses are manufactured and assembled identically, and the same bending moment is applied to each spoke. Because these conditions are very difficult to satisfy during fabrication and assembly, this paper assumes that a very small displacement *δ* occurs at the end of the cluster dither spoke owing to the externally applied bending moment and proposes the new boundary condition in Equation (44). The coefficients of Equation (43), obtained using the boundary conditions in Equations (9) and (44) and the boundary conditions in Equation (9), are expressed in Equation (45).

(37) $$E_{bp}I_{t}=E_{b}I_{b}+2E_{p}I_{p}$$

(38) $$I_{b}=\frac{ht_{b}^{3}}{12},\;I_{p}=\frac{ht_{p}^{3}}{12}+ht_{p}{\left(\frac{t_{b}+t_{p}}{2}\right)}^{2},\;I_{t}=\frac{h{\left(t_{b}+2t_{p}\right)}^{3}}{12}$$

(39) $$E_{bp}=\frac{E_{b}t_{b}^{3}+E_{p}\left(8t_{p}^{3}+12t_{p}^{2}t_{b}+6t_{p}t_{b}^{2}\right)}{t_{b}^{3}+8t_{p}^{3}+12t_{p}^{2}t_{b}+6t_{p}t_{b}^{2}}$$

(40) $$I_{i}=\frac{ht_{i}{\left(x\right)}^{3}}{12}$$

(41) $${\left(EI\right)}_{eq}=\frac{\int_{0}^{L_{1}}E_{b}I_{1}{\left(\frac{d^{2}Y(x)}{dx^{2}}\right)}^{2}dx+\int_{L_{1}}^{L_{2}}E_{b}I_{2}(x){\left(\frac{d^{2}Y(x)}{dx^{2}}\right)}^{2}dx+\int_{L_{2}}^{L_{3}}E_{b}I_{3}{\left(\frac{d^{2}Y(x)}{dx^{2}}\right)}^{2}dx+\int_{L_{3}}^{L_{4}}E_{bp}I_{4}{\left(\frac{d^{2}Y(x)}{dx^{2}}\right)}^{2}dx+\int_{L_{4}}^{L}E_{b}I_{5}{\left(\frac{d^{2}Y(x)}{dx^{2}}\right)}^{2}dx}{\int_{0}^{L}{\left(\frac{d^{2}Y(x)}{dx^{2}}\right)}^{2}dx}$$

(42) $${\left(\rho A\right)}_{eq}=\frac{\int_{0}^{L_{1}}m_{s1}(x)Y^{2}(x)dx+\int_{L_{1}}^{L_{2}}m_{s2}(x)Y^{2}(x)dx+\int_{L_{2}}^{L_{3}}m_{s3}(x)Y^{2}(x)dx+\int_{L_{3}}^{L_{4}}m_{s4}(x)Y^{2}(x)dx+\int_{L_{4}}^{L}m_{s5}(x)Y^{2}(x)dx}{\int_{0}^{L}Y^{2}(x)dx}$$

(43) $$Y(x)=A+Bx+Cx^{2}+Dx^{3}+Ex^{4}$$

(44) Y(L)=δ

(45) $$A=B=0,\;C=\frac{3\delta +3L^{4}}{2L^{2}},\;D=\frac{-\delta -5L^{4}}{2L^{3}},\;E=1$$

By substituting the mode shape function *Y*(*x*) of Equations (43) and (45) into Equations (41) and (42), obtaining the equivalent stiffness and equivalent mass per unit length of the cluster dither spoke, and substituting these values into Equation (23), the final natural frequency analysis of the cluster dither can be performed.

## 4. Cluster Dither Natural Frequency Design and Validation Through M&S

The natural frequency of a single-axis dither with a uniform and homogeneous spoke is basically obtained using Equation (8). However, Section 3 showed that Equation (23) should be used to analyze the natural frequency of a cluster dither with a non-uniform and heterogeneous spoke. This section presents the natural frequency design of the cluster dither and validation through M&S based on the analysis results for the cluster dither with partially tapered spokes described in Section 3.

Among the variables of Equation (23) required to calculate the natural frequency of the cluster dither, the equivalent stiffness (*EI*)*_eq_* and equivalent mass per unit length (*ρA*)*_eq_* are determined by the shape and material of the cluster dither spoke, whereas (*λL*)*_n_* is determined by the boundary conditions of the cluster dither. Therefore, the accuracy of Equation (23) depends on the accuracy with which its component variables are calculated.

As shown in Figure 5 and Figure 6, the cluster dither consists of three identical dither spokes, gyro mounting parts (*M*_1_ and *M*_2_), and RLGs (*M*_3_). In Equation (23), the dither spoke length *L* is determined considering the outer diameter of the IMU and the RLG arrangement. Because SUS304 was selected as the dither material, the equivalent mass per unit length (*ρA*)*_eq_* can be obtained by substituting the densities of SUS304 and the PZT and the mass formulas for the respective dither spoke length sections in Table 2 into Equation (42). The mode shape function *Y*(*x*), required to calculate the equivalent stiffness and equivalent mass per unit length, can be obtained using Equations (43) and (45). In Equation (44), the small displacement *δ* at the dither spoke end was introduced as an equivalent boundary-condition parameter to represent the effective compliance of the supported end, accounting for deviations from the ideal clamped–supported boundary condition in which the end displacement is zero, the translational stiffness at the dither end is infinite, and the rotational stiffness is zero. In the actual dither structure, such effective boundary flexibility may arise not only from the finite stiffness of the support and joint regions but also from assembly conditions. Therefore, *δ* is not an intrinsic material property of a specific material; rather, it is an equivalent structural-level parameter representing the effect of the flexibility of the end support on the dynamic response of the structure. Accordingly, a larger *δ* indicates a more flexible effective end boundary, whereas a smaller *δ* indicates a stiffer effective end boundary. For an exact analysis, the translational stiffness k_t_ and rotational stiffness *k_r_* at the dither spoke end should be determined accurately, incorporated into the end boundary conditions as shown in Equations (46) and (47), and then used to obtain λL. Because accurate values are difficult to obtain in practice, this study assumes that the dither spoke end undergoes only translational motion with a small displacement *δ*.

(46) $${\left(EI\right)}_{eq}\frac{\partial^{3}y(L,t)}{\partial x^{3}}-k_{t}y(L,t)-Md_{x}\frac{\partial^{3}y(L,t)}{\partial t^{2}\partial x}=0$$

(47) $${\left(EI\right)}_{eq}\frac{\partial^{2}y(L,t)}{\partial x^{2}}-k_{r}\frac{\partial y(L,t)}{\partial x}+J_{zz}\frac{\partial^{3}y(L,t)}{\partial t^{2}\partial x}=0$$

**Figure 9:**
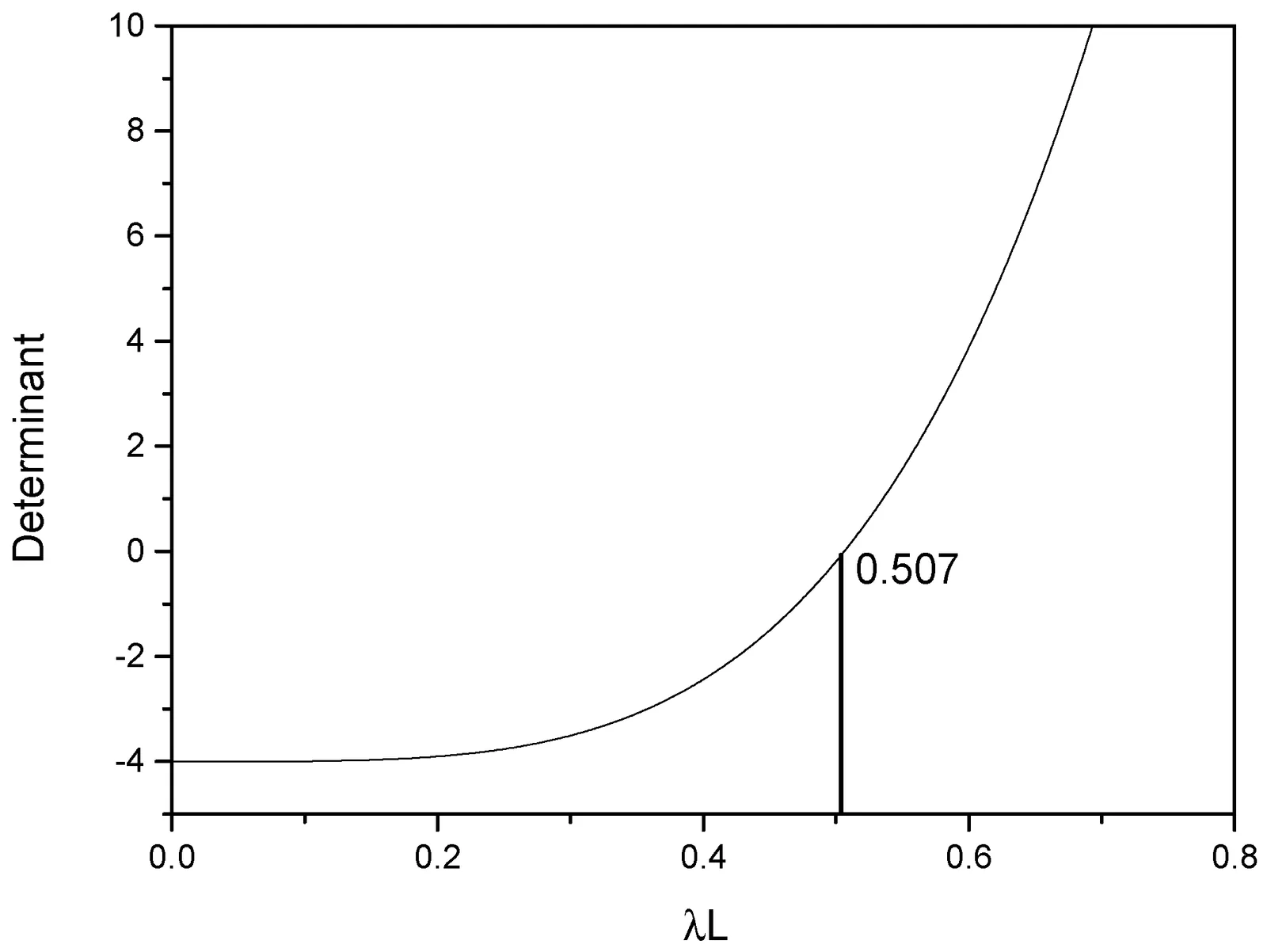
Determinant of Equation (29).

The coefficients *J_zz_* and *M* required to calculate the clamped–supported cantilever boundary conditions in Equations (27) and (28) are calculated using Equation (36), and the mass-related coefficient *M_dx_* can be calculated as *M_dx_* = *m_a_*_1_*d_x_*_1_ + *m_a_*_2_*d_x_*_2_ + *m_a_*_3_*d_x_*_3_, using the x-direction offsets of the respective models in Figure 6 and the masses of the respective models in Equations (30), (32) and (34). In addition, λL is determined by solving for the values where the determinant of Equation (29) vanishes. Figure 9 shows the simulation result for Equation (29), and the value of λL is 0.507. This value is used as the coefficient for calculating the first natural frequency in Equation (23).

The final design results of the cluster dither satisfying the natural frequency requirement of 800 Hz or higher are summarized in Table 3. Table 3 separately lists the design results for the dither spoke, gyro mounting parts, and RLG models that compose the cluster dither.

**Figure 10:**
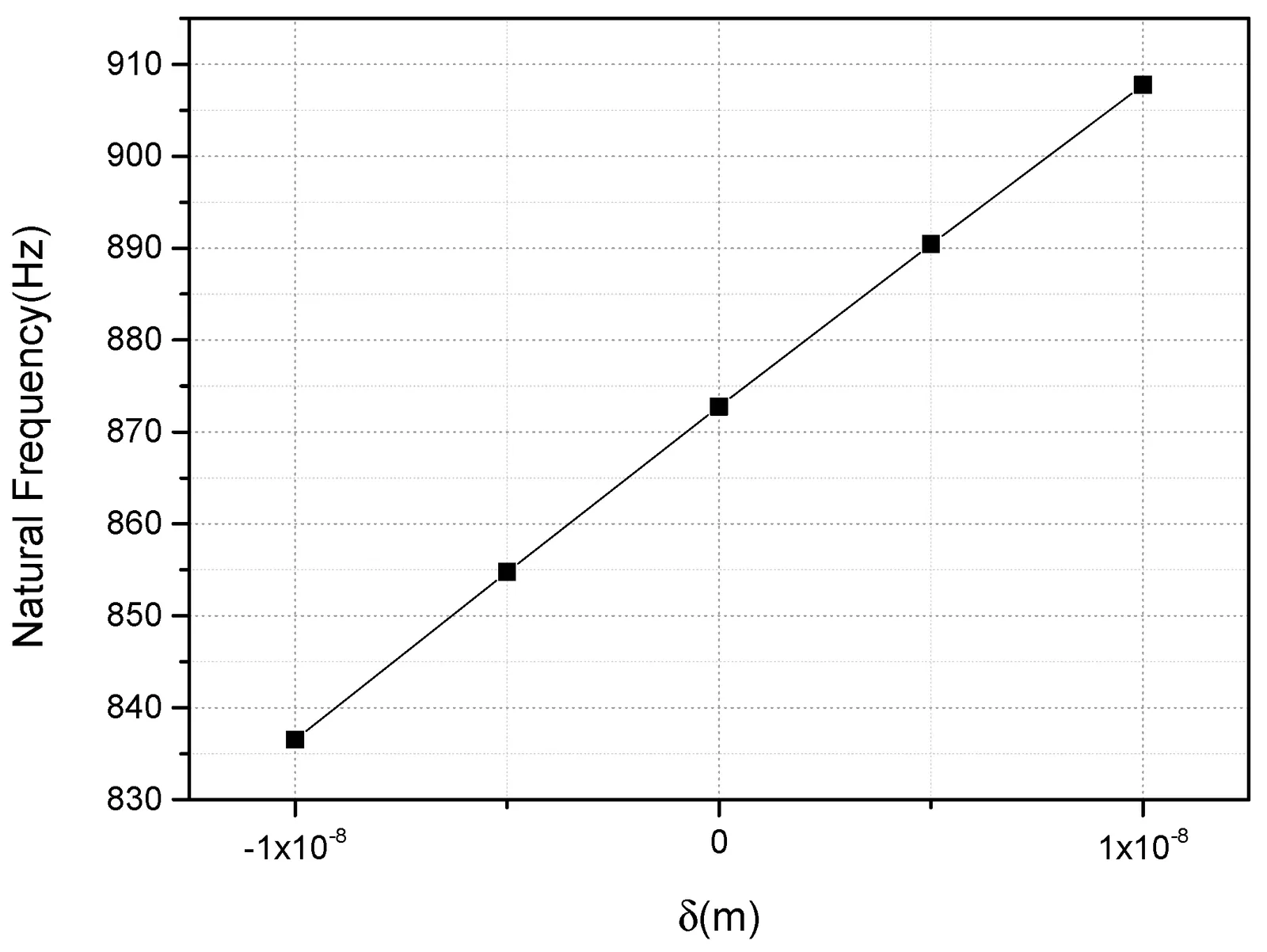
Natural frequency of the cluster dither according to δ in Equation (44).

Assuming that the small displacement *δ* at the dither spoke end lies within ±10 nm, the mode shape function *Y*(*x*) in Equations (43) and (45) is substituted into Equations (41) and (42) to obtain the equivalent stiffness and equivalent mass per unit length. These values, together with the dither spoke parameters in Table 3 and the λL value in Figure 9, are substituted into Equation (23); the resulting cluster dither natural frequency is plotted against *δ* in Figure 10. When *δ* is zero in Figure 10—that is, when the dither spoke end has no displacement—the cluster dither natural frequency is 872.75 Hz. The frequency decreases for displacement in the negative direction and increases for displacement in the positive direction. This behavior is consistent with the definition of *δ* as an equivalent boundary-condition variable representing the effective flexibility of the support. In Figure 10, *δ* represents the direction and magnitude of the displacement at the support; therefore, a change in *δ* corresponds to a change in the equivalent boundary condition at the support. Consequently, the equivalent end-support stiffness changes, resulting in a variation in the natural frequency. As mentioned above, *δ* in this study is not an independently measured physical parameter but rather a model variable introduced to equivalently represent the finite flexibility of the actual support. The range of ±10 nm was selected to account for the frequency variations observed under the actual experimental conditions.

**Figure 11:**
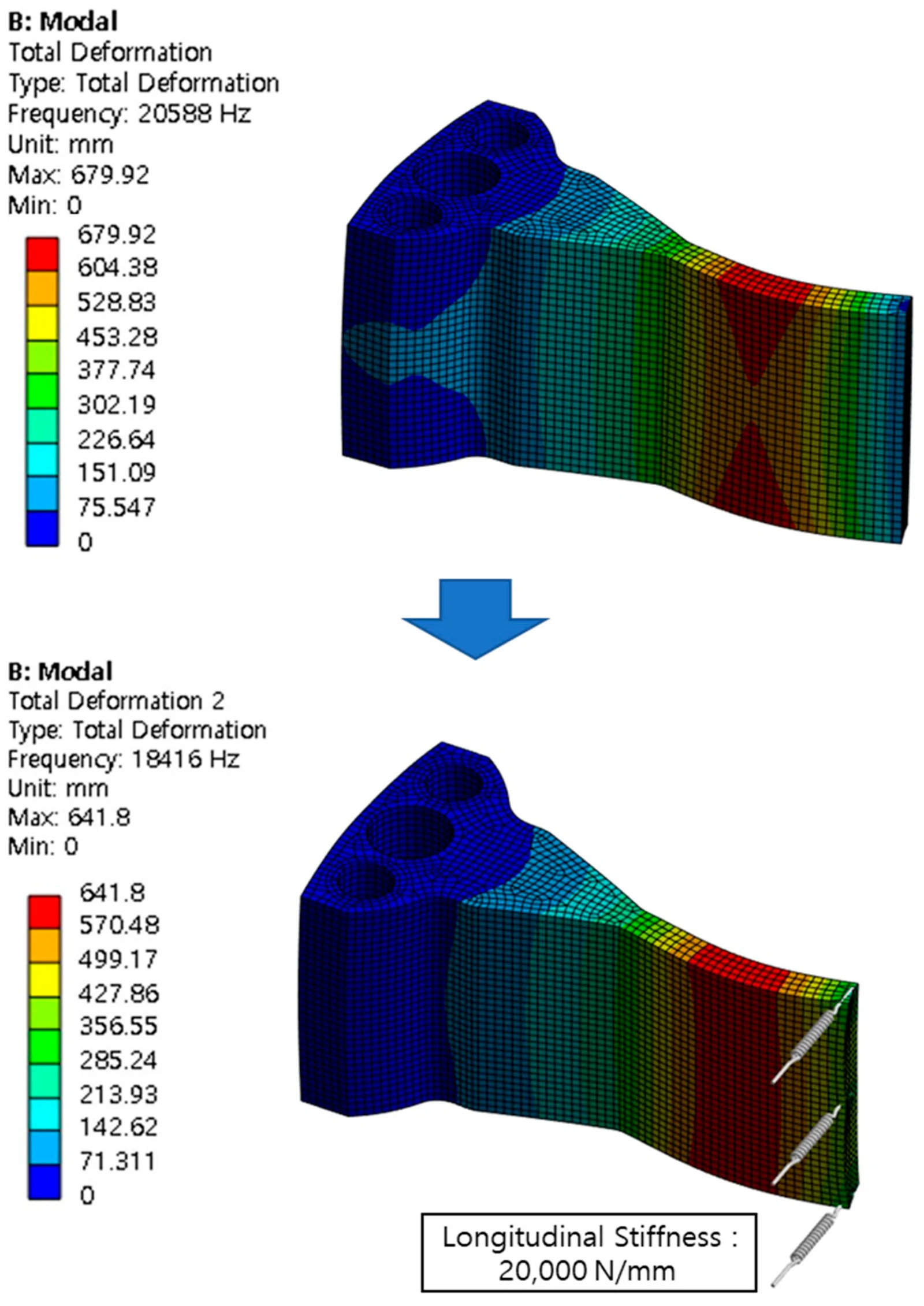
Effect of end-support stiffness on the natural frequency obtained from ANSYS.

To further examine the physical influence of the end boundary condition, an ANSYS analysis was performed in which only the end-support stiffness was varied while all other structural and material parameters were kept unchanged. As shown in Figure 11, the natural frequency was approximately 20.5 kHz under a relatively high end-support stiffness condition, whereas it decreased to approximately 18.4 kHz when the end-support stiffness was reduced to 20,000 N/mm. These results demonstrate that the end-support stiffness directly affects the natural frequency of the structure. As the end-support stiffness decreases, the effective boundary becomes more flexible, resulting in a reduction in the natural frequency. This behavior is consistent with the definition of *δ* as an equivalent boundary-condition variable representing the effective flexibility of the support. However, this analysis was conducted to independently examine the influence of the end boundary condition and was not intended to independently determine the absolute value of *δ*. Therefore, the analysis demonstrates that a change in the end-support stiffness can alter the effective boundary condition and thereby affect the natural frequency.

**Figure 12:**
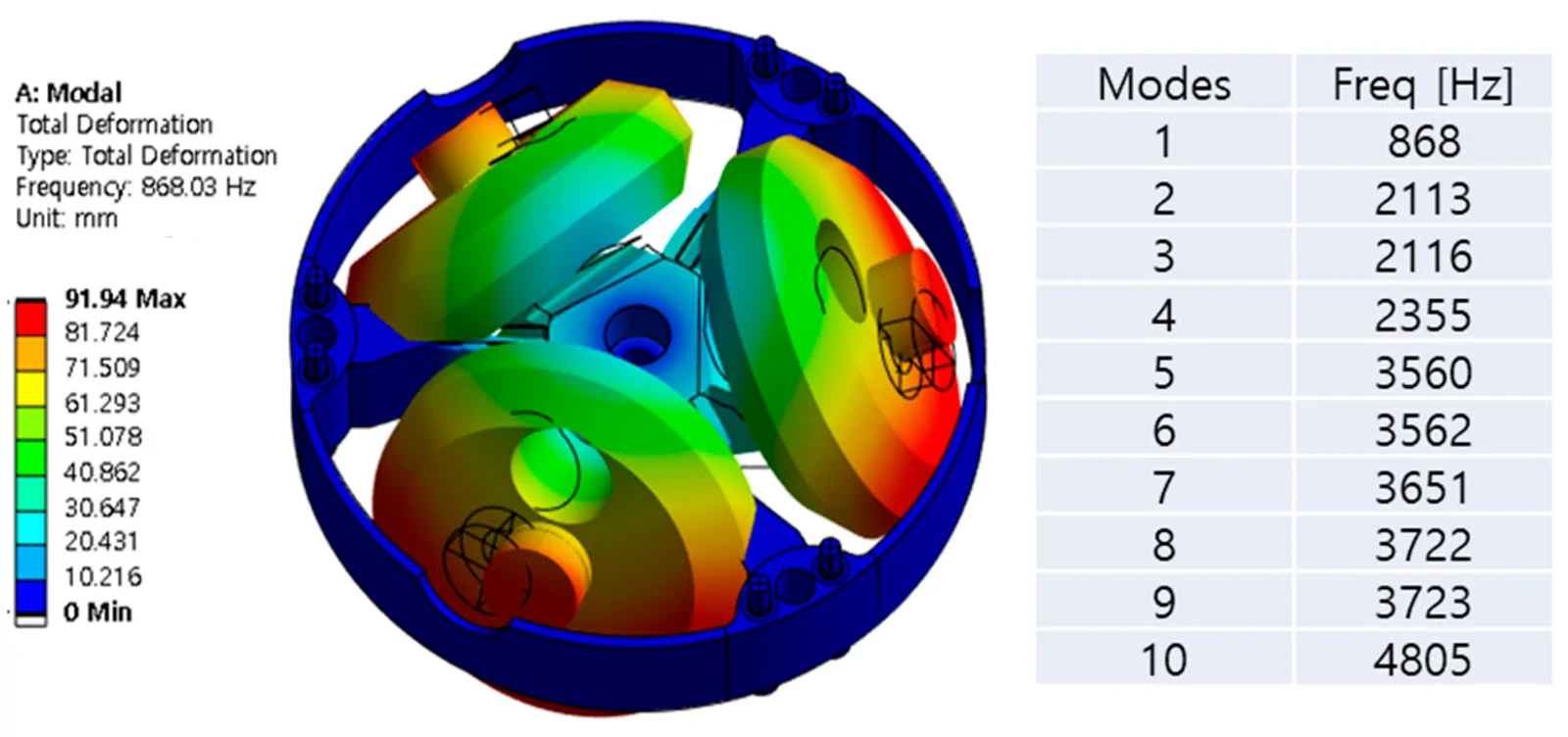
Modal analysis of the cluster dither.

To verify the analysis results in Figure 10, finite element analysis was performed using ANSYS as the M&S tool. For the analysis, each component was modeled to have the same structural characteristics as the components in Table 3. The ANSYS result is shown in Figure 12. In Figure 12, the circular structure is a dummy gyro body designed to have the same moment of inertia as the actual RLG. The final natural frequency obtained from the analysis was 868.03 Hz, which differs by approximately 4.72 Hz from the natural frequency of 872.75 Hz obtained by the theoretical analysis in Figure 10. This result corresponds to an error of approximately 0.5% between the theoretical analysis and M&S results, which is considerably more accurate than the approximately 2% error reported in Reference [6] for the natural frequency analysis of a single-axis dither. Therefore, the theoretical analysis results for the natural frequency of the cluster dither presented in this paper are considered valid.

As noted in the introduction, the dither is driven by applying to the PZT a sinusoidal voltage corresponding to the first-mode dither natural frequency. Therefore, even if natural frequency components of the second and higher modes exist, their angular rate amplitudes are expected to be very small and not to affect RLG performance. The higher-mode natural frequency analysis results for the dither are shown in Figure 12. The second-mode natural frequency is separated from the first-mode natural frequency by 1245 Hz and is therefore not expected to cause mutual interference. If sinusoidal motion at the first-mode natural frequency ω_1_ is applied to a dither resonating at the second-mode natural frequency ω_2_, the corresponding amplitude is reduced by the factor in Equation (48). Assuming a damping ratio of 0.01 in Equation (48), the angular amplitude component at the second-mode natural frequency decreases to approximately 0.98% of the first-mode angular amplitude. Using the method presented in Reference [2], the influence of the second-mode angular rate component on RLG random-walk performance was estimated to cause only an approximately 0.49% degradation. Thus, higher-mode natural frequency components do not significantly affect RLG performance.

(48) $$\tau =\frac{\zeta_{1}\omega_{2}^{2}}{\omega_{2}^{2}-\omega_{1}^{2}+\zeta_{2}\omega_{1}\omega_{2}}$$

**Figure 13:**
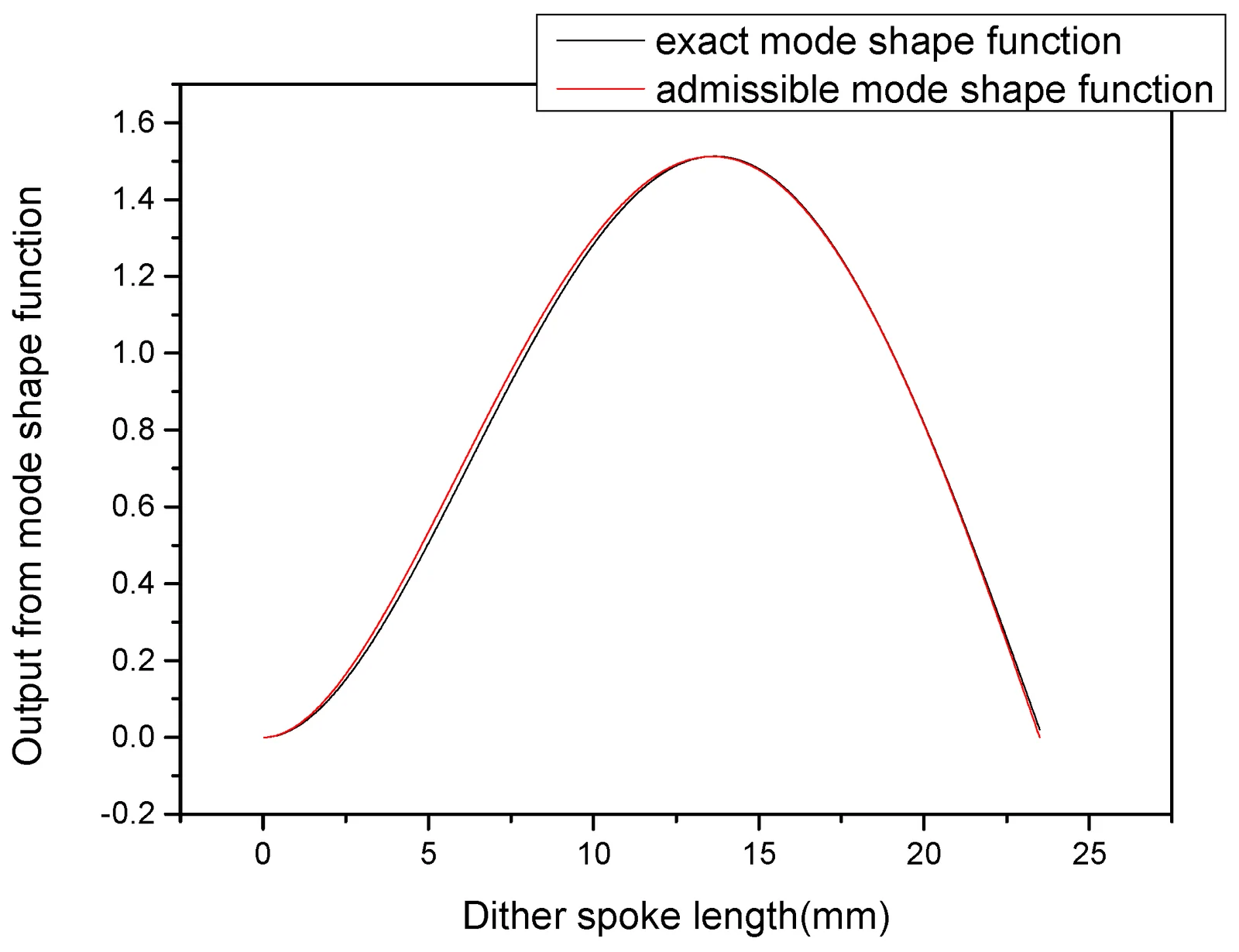
Comparison of exact and admissible mode shape functions.

To examine the natural frequency sensitivity of the admissible shape function in Equation (43), its modeling error was analyzed relative to the exact shape function in Equation (5). The coefficients of Equation (43) were obtained using Equation (45). For Equation (5), the relationship between c_1_ and c_3_ was obtained from the end boundary conditions of the dither spoke, and λ was calculated using λL_1_ in Table 1. After all coefficients were determined, the amplitude coefficients were adjusted so that Equations (5) and (43) had the same amplitude, and the two functions were compared along the dither spoke length, as shown in Figure 13. Figure 13 confirms that the two shape functions have nearly identical values. To analyze the natural frequency sensitivity of Equation (43), the dither spoke was assumed to be a uniform cantilever beam and Equation (22) was evaluated, yielding the shape function sensitivity equation in Equation (49). The values obtained by substituting Equations (5) and (43) into Equation (49) were then compared. The value calculated using Equation (5) was approximately 0.3% smaller than that calculated using Equation (43), demonstrating that using Equation (43) as the shape function has little effect on the natural frequency analysis. Because the natural frequency results in this paper differed from the test value by approximately 0.5%, use of Equation (5) might have improved the analytical accuracy to approximately 0.2%. The amplitude coefficients used to equalize the amplitudes of Equations (5) and (43) cancel during calculation of the equivalent stiffness and equivalent mass per unit length and therefore have no effect.

(49) $$\beta =\sqrt{\frac{\int_{0}^{L}{\left(\frac{d^{2}Y(x)}{dx^{2}}\right)}^{2}dx}{\int_{0}^{L}Y^{2}(x)dx}}$$

To compare the differences among the natural-frequency analysis methods, the results obtained using the conventional and proposed analytical methods were compared with the ANSYS results, as summarized in Table 4. As shown in Table 4, the methods presented in References [6,12] are based on simple cantilever-beam models and do not explicitly account for the end boundary condition of a single dither, resulting in very large errors in the predicted natural frequency. Similarly, when the conventional Rayleigh-based analysis method was directly applied to the cluster dither structure considered in this study, the natural frequency was significantly overestimated. In addition, even when the natural-frequency analysis method proposed in this paper was applied, omission of the shear-force equilibrium condition at the support resulted in an overestimated natural frequency. When both the moment equilibrium and shear-force equilibrium conditions at the support were properly applied, the calculated natural frequency was approximately 870 Hz, which agreed well with the ANSYS result. These comparisons indicate that the primary source of the large analytical errors is not the beam theory itself, but rather the inadequate representation of the dynamic boundary conditions at the support. They also show that, for reliable prediction of the natural frequency of the cluster dither, not only the partially tapered geometry and support boundary conditions but also the rotational inertia and offset of the end mass must be properly taken into account. As shown in Table 4, the stepwise reduction in the calculated natural frequency from 2465.9 Hz to 872.75 Hz demonstrates the importance and validity of properly incorporating the dynamic equilibrium conditions at the support in the natural-frequency analysis.

The large error observed in the conventional Rayleigh-based analysis is considered to arise not from an inherent limitation of the Rayleigh method itself, but rather from the difficulty of constructing an admissible trial function that simultaneously represents the actual dynamic mode shape of the present structure and satisfies the end equilibrium conditions. In particular, because the natural frequency is not known a priori for the present structure, the trial function must be assumed in advance, making it difficult to construct a shape function that simultaneously accounts for the partial taper, the inertia and offset of the end mass, and the moment and shear-force equilibrium conditions at the support. The conventional method remains valid as a beam-theory approach; however, its application to the present cluster dither requires a shape function that satisfies the dynamic end boundary conditions. In this study, this issue was addressed by explicitly imposing both the moment and shear-force equilibrium conditions.

## 5. Experimental Validation of the Natural Frequency Design of the Cluster Dither

**Figure 14:**
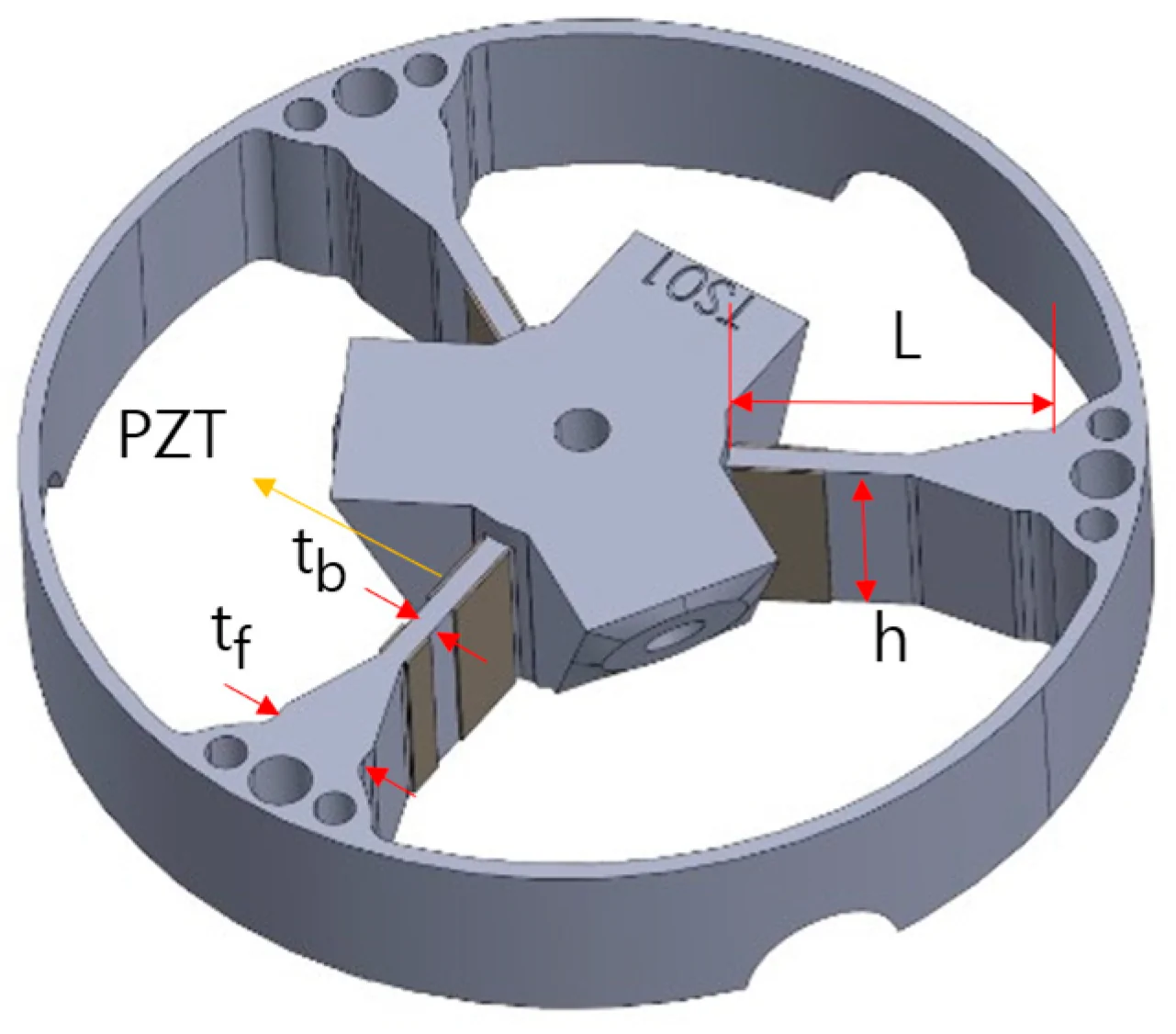
Design result for the cluster dither.

This section presents the test results obtained to verify the design validity and normal operation of the cluster dither designed in Section 3 and Section 4. The cluster dither designed in Section 3 and Section 4 was fabricated as shown in Figure 14. To confirm the natural frequency of the fabricated cluster dither, a dummy gyro body identical to that in Figure 12 was fabricated and mounted on the cluster dither, and impact-hammer tests and dither driving tests were performed.

An impact-hammer test was performed to confirm the performance of the fabricated cluster dither. Because a dither fixed to the IMU housing has a natural frequency different from the free–free condition owing to the stiffness introduced by fastening devices such as bolts, the impact-hammer test was performed to determine the change in natural frequency caused by bolt fastening. The experiment was conducted for two cases: the dither suspended in air (suspension method) and the dither attached to the IMU housing. For each case, the natural frequency of the dither was obtained using the impact-hammer test and compared with M&S results. The M&S results differed from the suspension-method results by less than 0.4%; however, in the fixed condition using the IMU, the difference ranged from 0.4% to 2.6% depending on the boundary conditions of the bolted connection. A 2.6% difference occurred when the dither through-holes were modeled as fixed supports, and the results became closer to those of the suspension method as additional fixed-support conditions, such as the bolt–head contact surfaces, were added. These experimental results were reflected in the M&S model of the dither, establishing a reliable design process that minimizes the analysis error of the dither natural frequency in the mounting environment inside the IMU housing. As a result, the experimentally established M&S model can predict the natural frequency of dithers with various design values within 0.4% of the experimental values. For the dither whose design was finalized in this way, the theoretical and experimental modal analysis results differed by only 0.35% (ANSYS: 868.03 Hz; impact-hammer test: 865.00 Hz).

**Figure 15:**
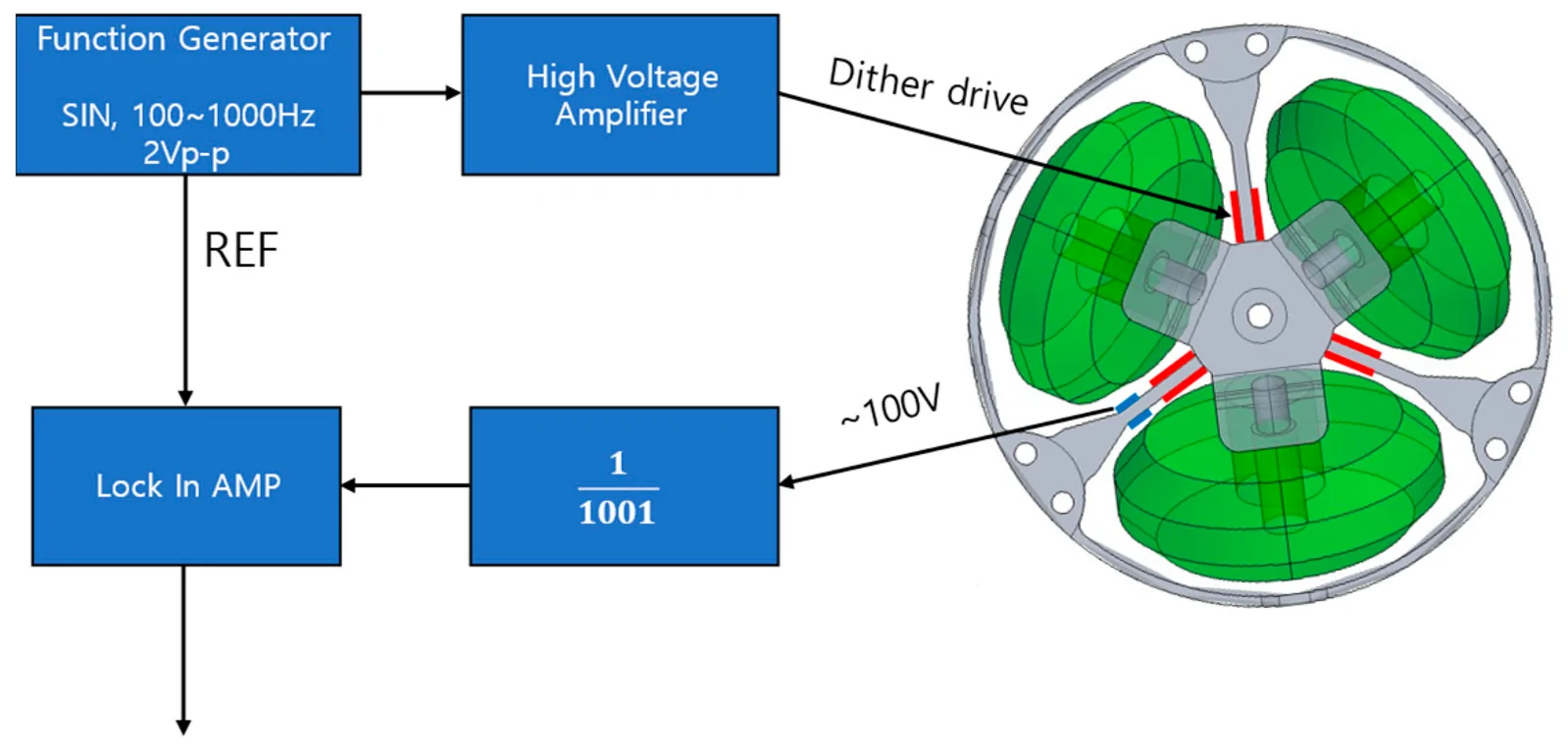
Test methodology for measuring the natural frequency of the cluster dither.

**Figure 16:**
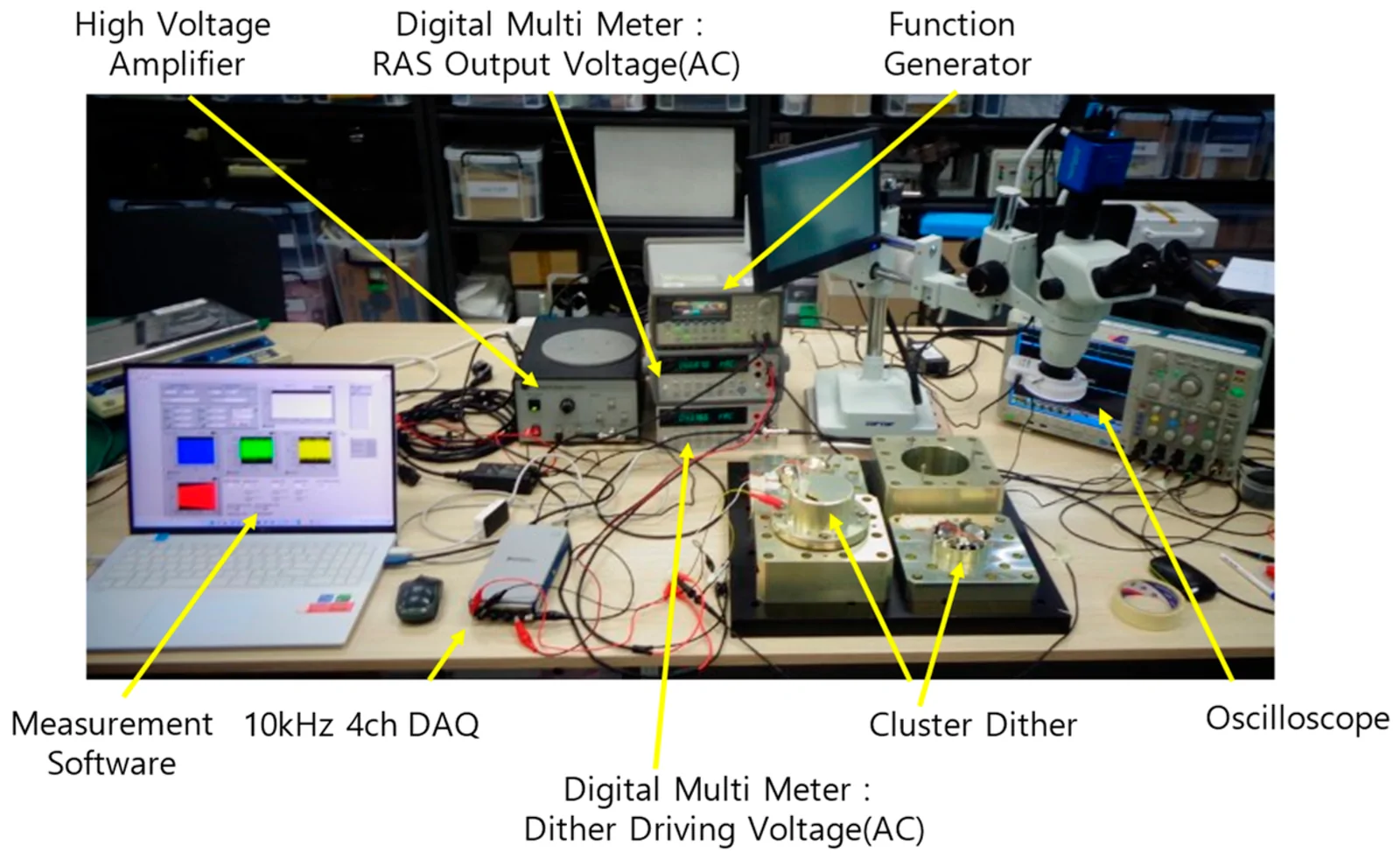
Experimental setup to confirm the natural frequency of the cluster dither.

A dither driving test was performed to confirm the performance of the fabricated cluster dither. The dither driving test method is illustrated in Figure 15. As shown in Figure 15, in addition to the three driving PZTs (red) attached to the dither spokes, the cluster dither has one sensing PZT (blue). The cluster dither was driven by applying sinusoidal voltages with frequencies from 100 Hz to 1000 Hz to the driving PZTs, and the natural frequency was confirmed by measuring the voltage output from the sensing PZT. The effect of PZT driving voltage on the natural frequency was also investigated by increasing the voltage applied to the driving PZT from 10 V to 140 V. The actual dither driving test setup is shown in Figure 16. In Figure 16, a function generator produces a sinusoidal signal of a specified frequency, and a high-voltage amplifier amplifies the signal to 10–140 V and applies it to the driving PZT. The driving PZT generates a bending moment in the dither spoke, and when the dither spoke undergoes sinusoidal motion, the sensing PZT outputs a voltage proportional to the bending of the spoke. The voltage applied to the driving PZT and the voltage output from the sensing PZT are measured by the digital multimeter shown in Figure 16; the measured values are transferred to a laptop through a data acquisition system, and the measurement software running on the laptop stores the measurements at a high sampling rate. The stored data were used to identify the point at which the phase difference between the sinusoidal signal applied to the driving PZT and the sinusoidal signal output from the sensing PZT was 90°, or the point at which the output voltage reached its maximum. The frequency at that point was determined as the natural frequency of the cluster dither.

**Figure 17:**
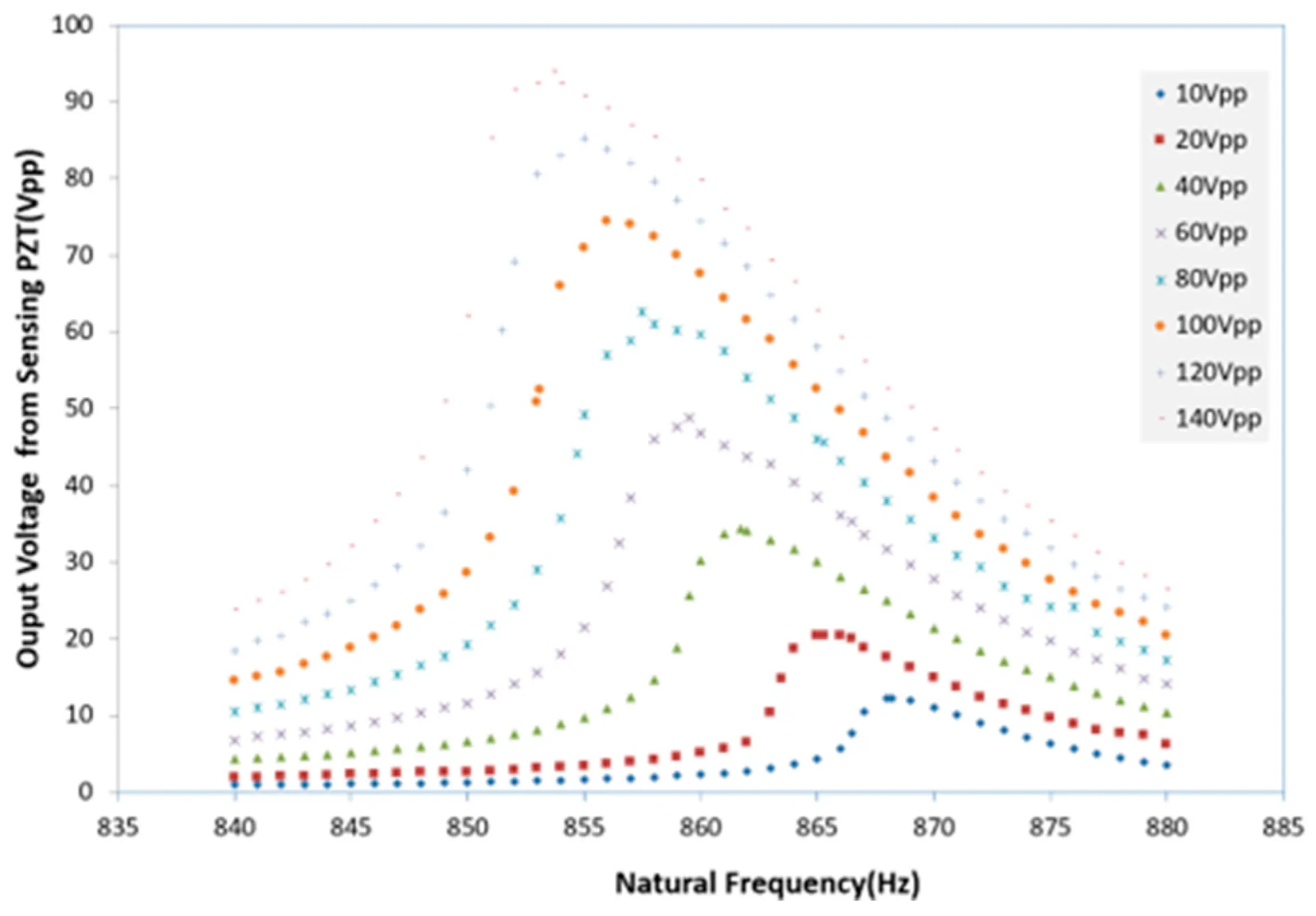
Sensing PZT output voltage according to PZT driving voltage.

**Figure 18:**
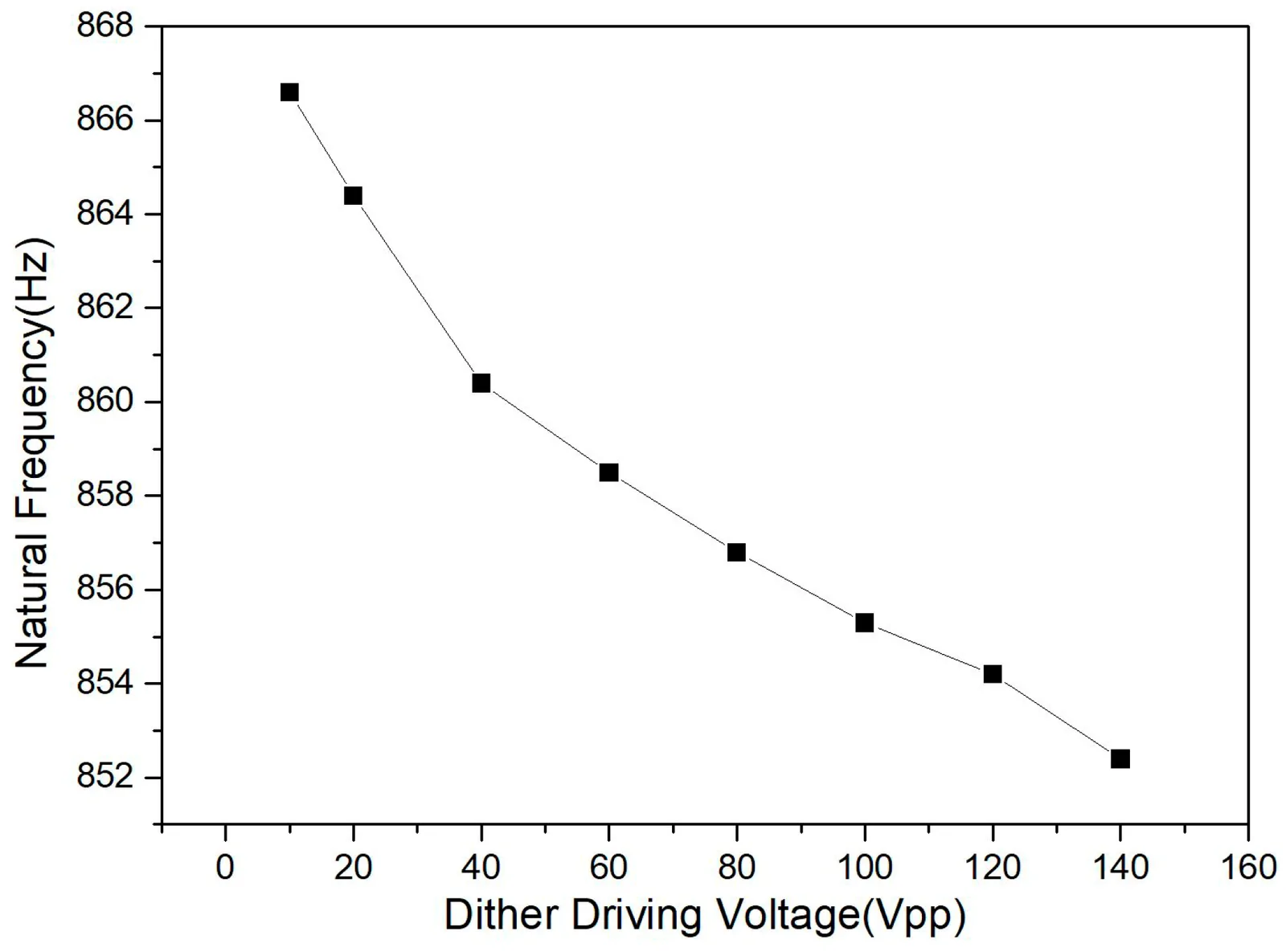
Natural frequency test results for the fabricated cluster dither.

Figure 17 shows the sensing PZT output voltage measured while varying the driving voltage and frequency. The measurement uses the natural frequency principle that the sensing PZT amplitude reaches its maximum when a voltage having the same frequency as the cluster dither natural frequency is applied to the PZT. Figure 17 also shows that the frequency at which the maximum amplitude occurs decreases as the PZT driving voltage increases. This behavior is similar to the frequency analysis results in Figure 10 and Figure 11, indicating that a small displacement occurs at the spoke end as the driving voltage increases and that the end translational stiffness consequently decreases. The frequencies at which the maximum amplitude occurs in Figure 17 are plotted against the voltage applied to the driving PZT in Figure 18. As shown in Figure 18, the dither natural frequency at a driving voltage of 10 V is 868 Hz, which is almost identical to the M&S result in Section 4. The test results in Figure 18 indicate that the end displacement is relatively small at low driving voltages. Therefore, the natural frequency of 872.75 Hz at *δ* = 0 in Figure 10 can be compared with the natural frequency measured at a driving voltage of 10 V in Figure 18. The difference is approximately 0.5%, experimentally confirming the validity of the natural frequency analysis presented in this paper. When the driving voltage is increased to 140 V, the dither natural frequency decreases to approximately 852 Hz, approximately 16 Hz lower than that at 10 V. This result shows that the dynamic characteristics of the structure can vary with the operating conditions. Although this observation alone does not permit the change in *δ* to be quantified directly, it is consistent with the analysis in Section 4 indicating that the effective boundary condition may change under different operating conditions.

When the same dither amplitude is maintained, the dither angular velocity is proportional to the natural frequency of the dither [6,10]. Compared with the 400 Hz natural frequency reported in Reference [6], the natural frequency obtained in this study is increased by a factor of 2.125; therefore, the dither angular velocity also increases by the same factor. In addition, because the Angle Random Walk (ARW) performance is inversely proportional to the square root of the dither angular velocity, this 2.125-fold increase in natural frequency reduces the ARW to approximately 69% of its original value under the same dither-amplitude condition. Therefore, assuming identical dither amplitude and driving conditions—and considering only the increase in angular velocity from the elevated natural frequency—the ARW could theoretically be improved by approximately 31%. However, the actual improvement in ARW in an RLG system may be affected by factors such as the Q-factor, PZT driving characteristics, and lock-in dynamics. These effects were not directly verified in this study, and system-level validation will be conducted in future work.

Based on the dither natural frequency analysis, additional practical analyses were performed to determine the frequency changes caused by mounting-angle errors and errors in the distance from the cluster dither end to the RLG center axis that can occur when an RLG is mounted on the cluster dither. In Equation (35), which is related to the RLG moment of inertia, mounting-angle errors of approximately 3° were applied to θ and ψ, and distance errors of approximately 5% were applied to d_x3_ and d_y3_. The resulting changes in natural frequency were then analyzed. The dither natural frequency decreased by 0.4% owing to the mounting-angle error and by 2.7% owing to the distance error. These decreases result from the increase in moment of inertia caused by the mounting-angle and distance errors. Thus, mounting errors arising during RLG installation do not substantially change the dither natural frequency. However, if the three RLGs mounted on the three dither spokes have different natural frequencies owing to mounting errors, the dither is driven at the natural frequency of the spoke carrying the sensing PZT. If the natural frequencies of the other two spokes differ from that of the spoke carrying the sensing PZT, their angular rate amplitudes are expected to be attenuated.

In addition to the geometric assembly errors examined above, the effects of variations in material properties on the natural frequency were also analyzed. For this purpose, the effects of changes in Young’s modulus and geometry were investigated for the dither material and the PZT over the operating temperature and applied-voltage ranges of the ring laser gyroscope. For the dither material, the variations in density and the thermal expansion coefficient over the operating temperature range are very small, and their effects on the natural frequency were therefore found to be negligible. In contrast, Young’s modulus has been reported to decrease by approximately 3% over the operating temperature range [21]. When this variation in Young’s modulus was taken into account, the natural frequency of the dither was found to decrease by approximately 1.5%. For the PZT attached to the dither spokes, the variations in density and the thermal expansion coefficient over the operating temperature range are also small, whereas Young’s modulus varies by several percent. However, the Young’s modulus of the PZT is affected more strongly by changes in the applied voltage than by temperature and has been reported to vary by up to approximately 20% over the operating voltage range [22]. When this variation in Young’s modulus was incorporated into Equation (39), the analysis showed that, because the Young’s modulus of the dither material is much higher than that of the PZT, even a 20% increase in the Young’s modulus of the PZT results in only an approximately 0.2% increase in the natural frequency of the dither. Meanwhile, a quantitative evaluation of the effects of factors such as machining errors, residual stresses, and preload conditions on the natural frequency would require statistical data obtained through repeated fabrication and measurement of individual dithers. Therefore, these factors were not analyzed within the scope of the present study and will be investigated in future work.

## 6. Conclusions

This paper presented a natural frequency analysis of a cluster dither with partially tapered spokes. Based on the conventional natural frequency analysis method for a single-axis dither with uniform and homogeneous spokes, this study showed that the spokes of a cluster dither with partially tapered, non-uniform and heterogeneous spokes can be appropriately modeled as clamped–supported cantilever beams. A method for deriving the equivalent stiffness and equivalent mass per unit length of the partially tapered, non-uniform and heterogeneous spoke was newly formulated, and a cluster dither natural frequency analysis method using these quantities was presented. In addition, the natural frequency analysis of the cluster dither with all three RLGs mounted was divided into four models: one dither spoke model, two models of the gyro mounting parts, and one RLG model. The end boundary conditions required for accurate natural frequency analysis were presented, and the importance of properly applying these conditions was confirmed. Using the natural frequency analysis method proposed in this paper, a cluster dither was designed to have a natural frequency of 800 Hz or higher. To verify the accuracy of the natural frequency analysis method proposed in this study, its analytical results were compared with those obtained using the methods presented in References [6,12] and the Rayleigh-based method. The comparison showed that the conventional methods and the Rayleigh-based method significantly overestimated the natural frequency, whereas the method proposed in this study showed good agreement with the M&S and experimental results. These results confirm that the proposed method is effective for the natural frequency analysis and design of the cluster dither.

To verify the accuracy of the natural frequency analysis for the cluster dither designed through theoretical analysis, the analytical result was compared with the natural frequencies obtained by M&S using ANSYS and by tests on the fabricated cluster dither. The comparison showed that the test results were most closely matched when the cluster dither was modeled and theoretically analyzed as a clamped–supported cantilever having a very small end displacement, rather than as a clamped–supported cantilever with exactly zero end displacement. The natural frequencies obtained by theoretical analysis, M&S, and testing differed from one another by less than approximately 0.5%, validating the natural frequency analysis method proposed in this paper. In addition, the cluster dither designed in this paper was confirmed to have a natural frequency of approximately 850 Hz or higher, satisfying the cluster dither requirement of 800 Hz or higher.

This study focused on the analysis and validation of the natural frequency of the cluster dither, and direct experimental verification of lock-in reduction and ARW improvement in an actual RLG system was not performed. Quantitative validation of these system-level performance improvements will be conducted in future studies using a complete RLG system.

## Figures and Tables

**Table 1:** Solution of characteristic equation.

Type of Beam	Solution of Characteristic Equation	(*λL*)_1_	Type of Dither
Clamped–free	coshλLcosλL=−1	0.596864π	Single-axis dither
Clamped–supported	tanλL−tanhλL=0	1.249876π	Cluster dither

**Table 2:** Equations related to dither spoke.

Length of Cantilever	Thickness	Mass
$0\leq x\leq L_{1}$	$t_{1}(x)=t_{f}$	$m_{s1}(x)=\rho_{b}t_{1}(x)h$
$L_{1}\leq x\leq L_{2}$	$t_{2}(x)=t_{f}+\frac{t_{b}-t_{f}}{L_{2}-L_{1}}(x-L_{1})$	$m_{s2}(x)=\rho_{b}t_{2}(x)h$
$L_{2}\leq x\leq L_{3}$	$t_{3}(x)=t_{b}$	$m_{s3}(x)=\rho_{b}t_{3}(x)h$
$L_{3}\leq x\leq L_{4}$	$t_{4}(x)=t_{b}+2t_{p}$	$m_{s4}(x)=\frac{\left(\rho_{b}t_{b}+2\rho_{p}t_{p}\right)}{t_{b}+2t_{p}}t_{4}(x)h$
$L_{4}\leq x\leq L$	$t_{5}(x)=t_{b}$	$m_{s5}(x)=\rho_{b}t_{5}(x)h$

**Table 3:** Parameter values of designed cluster dither model.

Type of Model	Parameters in Equations (30)–(35), (38) and (39) and Table 2						
Dither spoke	Parameter	L	h	m	t_b_	t_p_	t_f_
	Value	23.5 mm	15 mm	9.8 g	2 mm	0.3 mm	8 mm
	Parameter	L_1_	L_2_	L_3_	L_4_	E_p_	E_b_
	Value	1.0 mm	9.5 mm	15.5 mm	22.5 mm	65 GPa	190 GPa
M_1_	Parameter	J_zz1_	m_a1_	d_x1_	d_y1_	d_z1_	ψ
	Value	1.548 × 10^−5^	14 g	9 mm	3 mm	0	60°
M_2_	Parameter	J_zz2_	m_a2_	d_x2_	d_y2_	d_z2_	ψ
	Value	3.362 × 10^−5^	20 g	5 mm	10.5 mm	0	60°
M_3_	Parameter	J_zz3_	m_a3_	d_x3_	d_y3_	d_z3_	ψ/θ
	Value	4.858 × 10^−5^	71 g	1 mm	21 mm	9 mm	60°/64°

**Table 4:** Comparison of natural frequency predictions using different analytical approaches.

Analytical Approach	Boundary Condition	Natural Frequency [Hz]
Approach proposed in Reference [6]	Equation (5), Table 1	3234.6
	Equation (9), Table 1	14,184.6
Approach proposed in Reference [12]	Equations (5), (12) and (13)	1495.3
Conventional Rayleigh Method	Equations (9), (27) and (28)	2465.9
Approach proposed in this paper	Equations (9) and (28)	1811.5
	Equations (9), (27) and (28)	872.75
M&S (ANSYS)	Full Structural Model	868.03

## Data Availability

Dataset available on request from the authors.

## Abbreviations

The following abbreviations are used in this manuscript:

IMU	Inertial Measurement Unit
M&S	Modeling & Simulation
RLG	Ring Laser Gyroscope
PZT	Lead Zirconate Titanate
ARW	Angle Random Walk

## References

[1] Siouris G.M. (1993). Aerospace Avionics Systems, A Modern Synthesis.

[2] Aronowitz F. (1999). Fundamentals of the Ring Laser Gyro.

[3] Oelschlaeger J.M., Thielman L.O. (1990). GG1308 Ring Laser Inertial Measurement Systems-Honeywell’s Low Cost Solutions for Tactical Applications. IEEE Symposium on Position Location and Navigation.

[4] Vinnins M.F., Gallop L.D. (1993). Performance Evaluation of the Honeywell GG1308 Miniature Ring Laser Gyroscope.

[5] Killpatrick J. The GG1308-a Small Low-Cost RLG Electronic Gyroscope. Proceedings of the AIAA Astrodynamics Conference.

[6] Lee D.-C., Moon G., Lee J.-C. (2002). Mechanical Dither Design for Ring Laser Gyroscope. KSME Int. J..

[7] Lee D.-C., Shin D.-I., Kim Y.-S., Lee J.-Y., Han C.-S. (2009). Modelling the Mechanical Dither of a Ring Resonator. J. Mech. Eng. Sci..

[8] Li Y. (2013). Mechanical Design on Finite Element Method for Ring laser Gyroscope. Appl. Mech. Mater..

[9] Yu X., Wei G., Long X., Tang J. (2013). Finite Element Analysis and Optimization of Dither Mechanism in Dithered Ring Laser Gyroscope. Int. J. Precis. Eng. Manuf..

[10] Xiong Z., Yu X., Long X. (2016). Parametric Design for the Peak Amplitude of a Mechanical Dithering Ring Laser Gyroscope. Int. J. Appl. Electromagn. Mech..

[11] Hanse J.G. (1992). Cluster Dither Apparatus. U.S. Patent.

[12] Lei X., Wang Y., Wang X., Lin G., Shi S. (2021). Revisited on the Free Vibration of a Cantilever Beam with an Asymmetrically Attached Tip Mass. Math. Probl. Eng..

[13] Liu J., Weng J., Jiang J., Liu Y., Jiao M., Zhao K., Zheng Y. (2022). Study of the Steady-State Operation of a Dual-Longitudinal-Mode and Self-Biasing Laser Gyroscope. Sensors.

[14] Rao S. (2004). Mechanical Vibration.

[15] Timoshenko S.P., Young D.H., Weaver W. (1974). Vibration Problems in Engineering.

[16] Anderson G.L. (1978). Natural Frequencies of a Cantilever with an Asymmetrically Attached Tip Mass. AIAA J..

[17] Bhat R., Kulkarni M.A. (1976). Natural Frequencies of a Cantilever with Slender Tip Mass. AIAA J..

[18] Blevins R.D. (1979). Formulas for Natural Frequency and Mode Shape.

[19] Erturk A., Inman D.J. (2008). Issues in Mathematical Modeling of Piezoelectric Energy Harvesters. Smart Mater. Struct..

[20] Mochida Y., Eisenberger M. (2023). Choosing the Best Function for the Rayleigh-Ritz Vibration Analysis of Beams. EPI Int. J. Eng..

[21] British Stainless Steel Association (2026). Elevated Temperature Physical Properties of Stainless Steels.

[22] Zhang H., Yu J., Pei Y., Fang D. (2017). Butterfly Change in Electric Field-Dependent Young’s modulus: Bulge Test and Phase Field Model. J. Appl. Mech..

